# Zebrafish neuromast development: a target for endocrine disrupting chemicals?

**DOI:** 10.3389/ftox.2026.1733477

**Published:** 2026-03-17

**Authors:** Ellen Vandeputte, Evelyn Stinckens, Jade Verreth, Simone Fibiger Sørensen, Erik Fransen, Henrik Holbech, Lucia Vergauwen, Dries Knapen

**Affiliations:** 1 Zebrafishlab, Veterinary Physiology and Biochemistry, Department of Veterinary Sciences, University of Antwerp, Antwerpen, Belgium; 2 Department of Biology, University of Southern Denmark, Odense, Denmark; 3 Center of Medical Genetics, University of Antwerp and Antwerp University Hospital, Antwerpen, Belgium; 4 ECOSPHERE, Departement of Biology, University of Antwerp, Antwerpen, Belgium

**Keywords:** aquatic toxicity, developmental neurotoxicity, endocrine disruption, lateral line, neurosensory system, new approach methodologies (NAM), thyroid hormone system disruption, zebrafish embryo

## Abstract

In response to increasing evidence of human and environmental health impacts of endocrine disrupting chemicals (EDCs), screening and testing programs for EDC assessment are being developed, requiring characterization of potential adverse health effects. The development of the lateral line (LL), a neurosensory system in fish, has been suggested as a potential toxicological target of EDCs. The LL contains neuromasts with hair cells (HCs) which convert mechanical stimuli into neural signals. An exploratory study was performed to assess whether neuromast development is affected by a selection of model EDCs with different modes of action. Zebrafish embryos were exposed to EDCs, targeting estrogen and thyroid pathways, immediately after fertilization. The number of HCs in four neuromasts was counted at 120 h post fertilization. Methimazole and resorcinol (thyroid hormone [TH] synthesis inhibitors) elicited the strongest response, characterized by a reduction in HC numbers, while fulvestrant (anti-estrogen) slightly increased HC numbers. Further investigation confirmed a reduction of HCs and neuromasts after exposure to methimazole during late embryonic development, when TH synthesis is active. Gene transcript level analysis revealed a decreased marker for HC activity and increased markers of support cells, essential for HC regeneration. Taken together, neuromast development appears to be affected by certain EDCs in zebrafish embryos, at concentrations similar to those causing other effects (e.g., impaired swim bladder inflation). However, variability in the responses complicates characterization using the FM1-43 method. Further research, including rescue experiments and more sensitive or functionally relevant methods, is needed to clarify the mechanisms underlying EDC-induced HC disruption.

## 1 Introduction

In response to growing evidence of human and environmental health impacts of endocrine disrupting chemicals (EDCs), screening and testing programs for the assessment of EDCs are being developed and implemented throughout the world. A substance is considered an EDC if it causes an adverse effect, it has an endocrine mode of action, and the adverse effect is a consequence of the endocrine mode of action (WHO/UNEP, 2012). Ideally, test methods to assess EDCs are therefore capable of detecting perturbation of endocrine pathways of concern (i.e., endocrine mode of action, also referred to as endocrine activity) while at the same time providing information on potential adverse effects at the level of the organism (Matthiessen et al., 2017). Many of the currently existing test methods for the evaluation of potential ecotoxicological impacts of EDCs, and especially those capable of assessing adversity, require large numbers of animals (OECD, 2018). International initiatives, including the upcoming European Union’s roadmap to reduce animal testing, are promoting a shift toward alternative methods for chemical safety assessment, aiming to minimize reliance on animal data while maintaining robust safety evaluations (Cronin et al., 2025; European Chemicals Agency, 2023).

In this context, there is growing interest in the use of non-mammalian vertebrate embryos, especially fish and amphibian, which are not protected until the stage of independent feeding under some legislative frameworks, such as the European Union (EU) Directive 2010/63/EU on the protection of animals used for scientific purposes (Burden et al., 2022). Indeed, efforts to include endocrine disruption (ED)-sensitive endpoints in zebrafish embryo assays are ongoing and have been a priority in, e.g., OECD (Organization for Economic Cooperation and Development). This includes the development and validation of the EASZY assay for the detection of Endocrine Active Substances acting through estrogen receptors (ERs) using transgenic tg (cyp19a1b:GFP) Zebrafish embrYos (OECD Test Guideline [TG] 250, OECD (2021)). Another example is the ongoing validation of four endpoints (altered thyroid follicle morphology, altered thyroid hormone [TH] levels, impaired eye development, impaired swim bladder inflation), addressing both thyroid hormone system disruption (THSD) activity and adversity (Knapen et al., 2025), for inclusion in OECD TG 236 (Gölz et al., 2024a; Gölz et al., 2024b; Knapen et al., 2020; Stinckens et al., 2018; OECD, 2025). However, ED endpoints assessing adversity in fish early-life stages are generally non-specific, i.e., sensitive to but not diagnostic of ED in the context of regulatory frameworks. As a result, a suite of assays is likely needed and expanding the current set of endpoints remains a critical goal for improving regulatory ED assessment.

The development of the lateral line (LL) system, a neurosensory system in fish, has been suggested as a potential toxicological endpoint (Froehlicher et al., 2009b; Stengel et al., 2017). The LL system, which is necessary for prey detection, shoaling and rheotaxis, detects water movement and pressure gradients via specialized structures called neuromasts. They are positioned over the surface of the head (anterior LL, aLL) and body (posterior LL, pLL) and are innervated by axons extending from ganglia located in the head (Appel, 2013). A neuromast consists of sensory hair cells (HCs), support cells and mantle cells (Chitnis and Nogare, 2015). HCs detect water movement via deflection of stereocilia and convert these mechanical stimuli into neural signals (Holmgren et al., 2021). This endpoint is particularly relevant in light of the increasing attention to the link between EDCs and developmental neurotoxicity.

Tingaud-Sequeira et al. (2004) showed expression of three ER genes in neuromasts during zebrafish embryonic development and Lee et al. (2012) showed that exogenous estrogens effectively target ERs in the neuromasts. Moreover, Froehlicher et al. (2009a) found that morpholino knockdown of ERβ2 resulted in loss of HCs in zebrafish larvae. The estrogen pathway has also been shown to have a modulating effect on the vertebrate auditory system [reviewed in Caras (2013)], which is evolutionarily and developmentally related to the LL and also relies on the function of HCs (Alexandre and Ghysen, 1999).

Tissue-specific expression of TH receptors (TRs) has also been demonstrated in neuromasts of zebrafish embryos (Klein, 2023; Marelli et al., 2016). In surgeonfish, triiodothyronine (T3) treatment accelerated development of the trunk canal pores, structures similar to neuromasts in zebrafish, while a TH antagonist repressed their development (Besson et al., 2020). Hu et al. (2019) induced hypothyroidism (TH-) in zebrafish by conditional ablation of the thyroid follicles of transgenic fish with methimazole (MMI) exposure at 4–5 days post fertilization (dpf) and showed that TH- adults had 25% more total neuromasts than WT adults. Hu et al. (2019) further concluded that different parts of the LL respond differently to THs: THs inhibit neuromast proliferation in the head (aLL) but cause expansion of the neuromast population in the trunk (pLL).

Although studies linking EDC exposure to HC disruption and subsequent behavioral changes remain limited, emerging evidence suggests a functional connection. Nasri et al. (2021) demonstrated that exposure to 17α-ethinylestradiol (EE2) impairs HC regeneration and alters larval swimming behavior, characterized by prolonged inactivity, reduced time to cover long distances, and increased central arena occupancy. More broadly, toxicant-induced HC damage has been associated to behavioral alterations such as rheotaxis. While LL ablation reduces rheotactic and flow-related behaviors in zebrafish, it does not eliminate them entirely (Todd et al., 2017). Dose-dependent cisplatin exposure impairs HC integrity and reduces rheotactic performance (Niihori et al., 2015; Todd et al., 2017). Similarly, Newton et al. (2023) further showed that copper-induced HC ablation alters rheotactic patterns, with larvae swimming farther, for shorter durations, and with greater angular variance, yet retaining the basic rheotactic behavior. Collectively, these findings indicate that HC disruption affects LL-mediated behaviors and may serve as an endpoint for assessing neurotoxic effects.

Since existing knowledge suggests that EDCs can interfere with normal neuromast development, we conducted an exploratory study to assess whether neuromast development is affected by exposure to a selection of model EDCs with different modes of action. Zebrafish embryos were exposed to six known EDCs, including compounds targeting the estrogen and thyroid pathways, and functional HCs were counted at five dpf. Follow-up experiments were conducted aiming at a preliminary exploration of underlying mechanisms.

## 2 Materials and methods

### 2.1 Fish housing and egg production

Details on zebrafish (outbred wild type) broodstock housing and egg production can be found in Supplementary Material (SI) Section 1.1. Embryos were collected from a breeding group consisting of two females and three males. Zebrafish embryos until 120 h post fertilization (hpf), used in the experiments, are not protected under EU Directive 2010/63/EU.

### 2.2 EDC screening using acute zebrafish embryo toxicity test (ZFET)

Zebrafish embryos were exposed to seven chemicals in separate experiments: copper sulfate pentahydrate (CuSO_4_·5H_2_O, hereafter referred to as CuSO_4_) as a positive control; EE2, an ER agonist; fulvestrant (FUL), an ER antagonist; β-naphthoflavone (BNF), an aryl hydrocarbon receptor (AhR) agonist; methimazole (MMI), a thyroperoxidase (TPO) inhibitor; resorcinol (RSC), a TPO inhibitor, TR antagonist and transthyretin (TTR) binding inhibitor (Van Dingenen et al., 2024) and iopanoic acid (IOP), an iodothyronine deiodinase (DIO) inhibitor. Information on the preparation of stock solutions can be found in SI section 1.2 and 1.3. Exposure solutions were made fresh daily using reconstituted freshwater (same as for housing of adults) for daily medium renewal. pH was adjusted to 7.5 ± 0.1 using 1N HCl or 30 g/L NaHCO_3_ when required and conductivity stayed consistent at 500 ± 15 µS. Previous experiments in our laboratory under similar exposure conditions reported measured medium concentrations of 80%–100% for CuSO_4_ (Majid et al., 2025), 95%–103% for MMI (Stinckens et al., 2020), 81%–104% for RSC (Van Dingenen et al., 2024), 60%–80% for EE2 (Michiels, 2019; Michiels et al., 2019) and 84%–110% for IOP (Stinckens et al., 2020). No results on analytical verification were available for FUL and BNF. Final concentrations of EDCs were based on data previously obtained in our laboratory under similar experimental conditions (data not shown), ensuring all maximum concentrations remained below the LC20.

ZFETs were based on OECD TG 236 (OECD, 2025) with adaptations including extension of the exposure period, from <1 hpf and until 120 hpf (Supplementary Figure S2), and incubation at 28.5 °C ± 0.2 °C, as described by (Stinckens et al., 2018). Embryos were sequentially immersed in two volumes of exposure solution before plating. Embryos were individually placed in pre-saturated 24-well plates (2 mL medium/well; sterile cell culture plate, Sarstedt AG&Co, Nümbrecht, Germany) with one plate per concentration (20 embryos/concentration, four internal negative controls). Plates were sealed with parafilm (Parafilm®, Bemis Europe, Soignies, Belgium) and lids. Details on positive, negative and solvent controls can be found in SI section 1.4. Solvent controls were included when required: 0.01% for EE2 and 0.1% for FUL and BNF. These concentrations are not toxic to HCs [Supplementary Figure S1, Uribe et al. (2013)]. However, Uribe et al. (2013) observed a synergistic effect between DMSO and cisplatin but not with neomycin. The possibility of such solvent-chemical interactions cannot be fully excluded. Incubation conditions were 28.5 °C ± 0.2 °C with a 14/10 h light/dark cycle (MIR-254-PE, Panasonic, TCPS, Rotselaar, Belgium). Chorions of MMI-exposed embryos were manually removed at 96 hpf for eleutheroembryo (hatched but not yet exogenously feeding) analysis at 120 hpf to avoid impacts of impaired hatching on morphological observations. Each chemical was tested in duplicate on consecutive days.

### 2.3 Exposure window assessment of thyroid hormone system disruption

Three exposure windows, 0–72 hpf, 72–120 hpf and 0–120 hpf, were selected based on TH system development. ZFETs were conducted as above, using two sequential transfers in the respective medium, in order to start and end exposure windows. A sublethal concentration of 300 mg/L MMI was selected based on the EDC screening results (Section 3.2). Because of the short exposure window, higher concentrations (400 and 500 mg/L MMI) were also included for the 72–120 hpf window, the main window of interest.

### 2.4 Morphological assessment

Morphological assessments were conducted using a stereomicroscope (Leica S8APO, Leica Microsystems GmbH, Germany). Four apical endpoints for lethality (coagulation, absence of somite formation, non-detachment of the tail and lack of heartbeat) and hatching were evaluated every 24 h as indicators of lethality (OECD, 2025). At 120 hpf, sublethal morphological effects were additionally scored using a binary scoring system.

Eleutheroembryos were anesthetized in 0.1 g/L ethyl 3-aminobenzoate methanesulfonate (MS-222, CAS: 886–86–2, Sigma-Aldrich, Saint-Louis, United States, 98%), buffered to pH 7.5 using NaHCO_3_, then transferred to a carrier glass and embedded in 3% methyl cellulose. Twelve eleutheroembryos per treatment were photographed (Canon EOS 600D, 18 megapixels), using a calibrator to measure larval length. Images were processed with ImageJ (v1.53e, https://imagej.net/ij/).

### 2.5 Assessment of neuromast development

**FIGURE 1:**
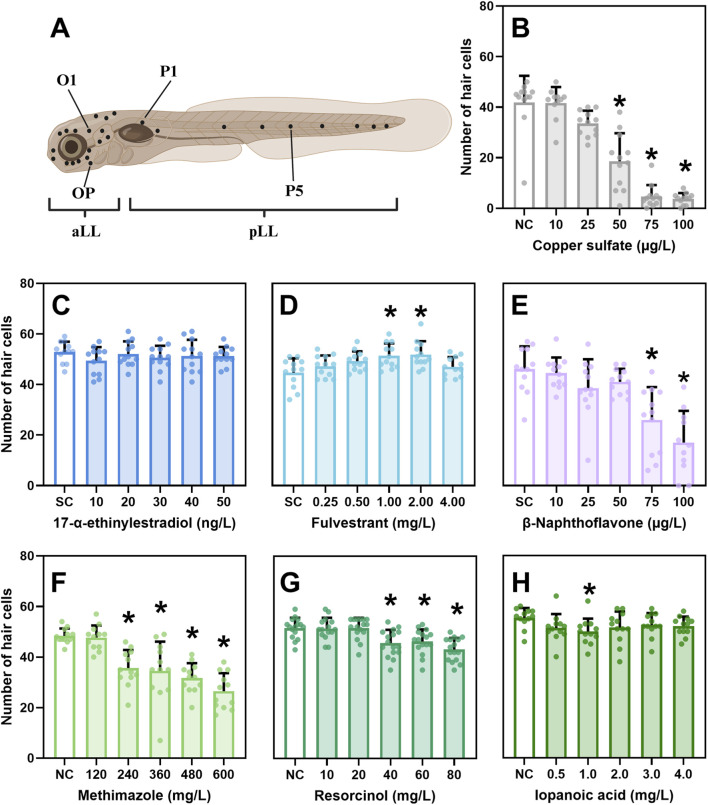
Effect of positive control and endocrine disrupting chemicals (EDCs) on the number of hair cells (HCs). **(A)** Stereotyped positions of anterior lateral line (aLL) and posterior LL (pLL) neuromasts in 120 h post fertilization (hpf) zebrafish larvae [developed in Biorender, adapted from Harris et al. (2003)]. The position names of neuromasts analyzed in this study are given: O = otic, OP = opercular, P = posterior. **(B–H)** Results of the number of HCs after exposure to **(B)** Copper sulfate, **(C)** 17-α-ethynilestradiol, **(D)** Fulvestrant, **(E)** β-naphthoflavone, **(F)** methimazole, **(G)** resorcinol, **(H)** iopanoic acid until 120 hpf. HCs were counted in four neuromasts and averaged. Supplementary Figure S2 shows the HCs counts per neuromast. Data are represented as mean ± standard deviation (SD). Sample size = 11–12. Statistical difference (p < 0.05) between negative control (NC) or solvent control (SC) and test concentration is denoted with*.

After 5 days of exposure, functional, mechanotransducive HCs in neuromasts were labelled by incubating the eleutheroembryos in 3 μM N-(3-Triethylammoniumpropyl)-4-(4-(Dibutylamino) Styryl) Pyridinium Dibromide (FM1-43, Invitrogen™, Waltham, United States) for 45s, followed by four 45s rinses with reconstituted fresh water in the dark (Supplementary Figure S2, SI section 1.5) [adapted from Santos et al. (2006)]. FM1-43 was selected since it is a widely used fluorescent dye that does not require specialized procedures or equipment beyond a fluorescence microscope. Importantly, FM1-43 selectively labels sensory HCs, as it is unable to penetrate the lipid bilayer of most cells but is internalized through endocytosis in HCs. Eleutheroembryos were briefly anaesthetized with buffered 0.2 g/L MS-222, positioned on their right flank in 3% methyl cellulose on a carrier glass and imaged using an Olympus IX71 inverted fluorescence microscope (Olympus Corporation, Shinjuku, Tokyo, Japan) with a FITC filter. Four neuromasts were analyzed per eleutheroembryo–two from the aLL (O1 and OP) and two from the pLL (P1 and P5) (Figure 1A). For preliminary EDC screening, 12 eleutherembryos per concentration were assessed. For time window experiments, 16–20 eleutherembryos were analyzed per concentration. In addition to HC counts, in the time window experiments, neuromast numbers in the aLL and pLL were counted. All counts were completed within 2.5 h to minimize variability and HC regeneration. HCs and neuromasts were counted manually and by the same, blinded, individual to eliminate experimenter’s bias.

### 2.6 Reverse transcription quantitative PCR analysis

Gene transcript analysis of genes related to neuromast development and *tpo,* as a marker for TPO inhibition, was performed on heads and bodies of eleutheroembryos exposed to 500 mg/L MMI in the 72–120 hpf window. Details on the sampling, the reverse transcription quantitative PCR (RT-qPCR) protocols, including primer sequences, can be found in SI section 1.6 and Supplementary Table S2.

### 2.7 Data analysis

All statistical analyses were performed in R (v. 4.5.0) (R Core Team, 2025) unless stated otherwise, with statistical significance defined as p < 0.05. Details on statistical analysis can be found in SI section 1.7.

## 3 Results

### 3.1 Morphological analysis

No mortality was observed in eleutheroembryos exposed to CuSO_4_, EE2, FUL, MMI, RSC and IOP, whereas treatment with 75 and 100 μg/L BNF resulted in 57.5% and 52.5% mortality, respectively (Supplementary Figure S3; Supplementary Table S3). Exposure to CuSO_4_ at the three highest test concentrations led to reduced swim bladder inflation (67.5%, 100% and 100%, Supplementary Table S3), reduction in larval length and delay in hatching (Supplementary Table S3). Morphological alterations were not observed after EE2 exposure. FUL exposure did not lead to any malformations. Exposure to BNF caused multiple malformations: cardiovascular malformations, head malformations and non-inflated swim bladders. MMI induced malformations, such as curvature of the spine and malformation of the head, at higher test concentrations (360–600 mg/L). Additionally, MMI delayed hatching, significantly reduced larval length (600 mg/L) and caused reduced swim bladder inflation. After RSC exposure, head malformations and impaired swim bladder inflation were observed. Reduced swim bladder inflation was observed after IOP exposure (4 mg/L).

Exposure to 300 mg/L MMI during time window experiments resulted in reduced swim bladder inflation during the continuous exposure (67.5%). In the 72–120 hpf window, swim bladder inflation was reduced by 32.5% after exposure to 500 mg/L MMI (Supplementary Table S4).

### 3.2 Initial screening: effect of EDC exposure on hair cells

The positive control, CuSO_4_, resulted in a significant decline in the number of HCs (Figure 1B), which validates the assessment method. EE2 exposure did not result in any effect on the number of HCs (Figure 1C). FUL exposure caused a significant increase in numbers of HCs in the 1 and 2 mg/L exposures (Figure 1D). BNF caused a reduction in the number of HCs in the two highest test concentrations that also caused mortality (Figure 1E). MMI caused a significant decrease in number of HCs starting from the 240 mg/L exposure (Figure 1F). RSC exposure also resulted in a significant reduction in the number of HCs starting from 40 mg/L (Figure 1G). After IOP exposure, a reduction of HCs was only observed in the 1 mg/L exposure (Figure 1H). Detailed data on the number of HCs for each of the four neuromasts can be found in Supplementary Figure S4. Additionally, effect size, confidence interval (CI) and p-values can be found in Supplementary Table S5. For both CuSO_4_ and MMI, a significant interaction was observed between test concentration and neuromast type. In the case of MMI, comparisons further revealed a significant interaction between the aLL and pLL (data not shown).

### 3.3 Effect of methimazole on neuromasts during specific time windows

**FIGURE 2:**
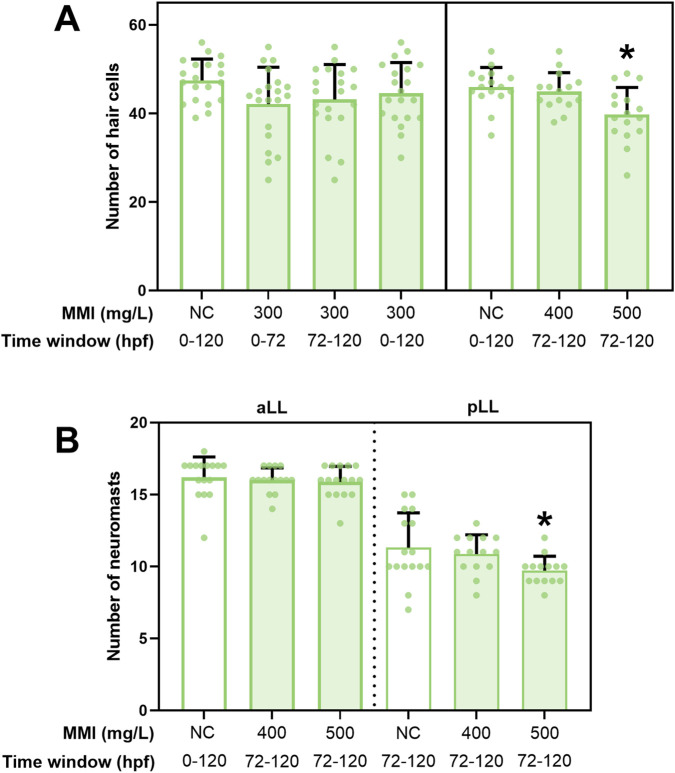
Effect of MMI exposure during different time windows on the total number of hair cells (HCs) and neuromasts at 120 h post fertilization (hpf). **(A)** Three different time windows were examined: 0–72 hpf, 72–120 hpf and 0–120 hpf. Counts of HCs of four different neuromasts have been summed. Supplementary Figure S3 shows the HCs counts per neuromast. **(B)** Effect of exposure to 400 and 500 mg/L MMI during 72–120 hpf time window on the total number of neuromasts at 120 hpf. The neuromasts of the anterior lateral line (aLL) and posterior lateral line (pLL) were counted separately. Data are represented as mean ± standard deviation (SD). Sample size = 16–20. Statistical difference (p < 0.05) between negative control (NC) and test concentration is denoted with*.

Exposure to 300 mg/L MMI from 0 to 72 hpf, 72–120 hpf or 0–120 hpf did not result in significant effects across the four neuromasts (Figure 2A; Supplementary Table S6). Exposure to 300 mg/L MMI led to a significant decline in the number of HCs in one neuromast (O1) after exposure from 0 to 120 hpf (Supplementary Figure S5A). A higher concentration (500 mg/L) during the 72–120 hpf time window led to a significant reduction in the sum of the number of HCs across the four neuromasts, in the absence of any mortality or gross malformations (only reduced swim bladder inflation was observed) (Figure 2A; Supplementary Table S7). The number of neuromasts of the aLL and pLL was also counted following exposure to 400 and 500 mg/L MMI from 72 to 120 hpf (Figure 2B). A reduced number of neuromasts was observed in the pLL after exposure to 500 mg/L MMI (Supplementary Table S8). The number of neuromasts was not correlated to larval length (p = 0.72 for aLL and p = 0.551 for pLL, Poisson regression, data not shown).

### 3.4 Gene transcript level analysis

**FIGURE 3:**
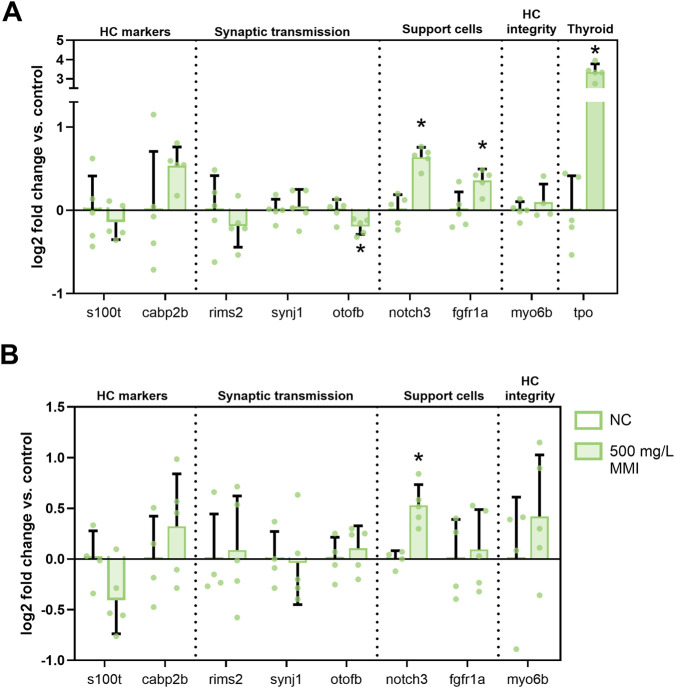
Relative gene transcript levels of neuromast and thyroid relevant genes after exposure to 500 mg/L MMI from 72 to 120 h post fertilization (hpf). Transcript levels were normalized using the geometric mean of two reference genes (*actb1* and *rpn2*). Five independent biological replicates of 20 larvae each were microdissected at 120 h post fertilization (hpf) to compare head [**(A)**, anterior LL] and body [**(B)**, posterior LL] samples. *Tpo* analysis was only performed on head samples due the localized presence of thyroid follicles in this region. *S100t* = S100 calcium binding protein T, *cabp2b* = calcium binding protein 2b, *rims2* = regulating synaptic membrane exocytosis 2, *synj1* = synaptojanin 1, *otofb* = otoferlin b, *Notch3* = notch receptor 3, *fgfr1a* = fibroblast growth factor receptor 1a, *myo6b* = myosin VIb. Data are represented as mean ± standard deviation (SD), with the left bar showing the negative controls (NC) and the right bar showing the 500 mg/L exposure for each gene. Statistical difference from NC: *p < 0.05.

MMI exposure (500 mg/L, 72–120 hpf) significantly decreased *otofb* (marker of synaptic transmission) in the head samples (Figure 3A). Additionally, a significant upregulation of support cell markers *notch3* and *fgfr1a* was observed, as well as increased *tpo* transcripts. In body samples, only *notch3* was significantly upregulated (Figure 3B). Primer efficiencies for *notch3* and *tpo* were 119.81% and 132.36% respectively, which may have caused some uncertainty in the fold change values.

## 4 Discussion

### 4.1 Fulvestrant increased the number of hair cells in neuromasts

To date, the impact of EE2 (an ER agonist) on HC development has not been directly investigated. However, Froehlicher et al. (2009a) demonstrate that ERβ2 is essential for neuromast development in zebrafish via an ERβ2 morpholine knockdown model. Based on these knockdown data, it was hypothesized that EE2 exposure would lead to an increase in HCs (Froehlicher et al., 2009a). However, EE2 exposure had no effect on HC number in the present study (Figure 1C). Nasri et al. (2021) reported that EE2 inhibited HC regeneration following CuSO_4_-induced ablation at seven dpf, with assessments conducted 24 and 48 h post-injury. As their study did not evaluate EE2 effects in the absence of prior damage, direct comparison with our continuous exposure model (<1 hpf to 120 hpf) is difficult. To put our results into perspective, we consider previously reported toxicological thresholds for EE2 across different endpoints. Estrogenic activity of EE2 has been demonstrated across multiple transgenic zebrafish models. Induction of mCherry expression in a vitellogenin (an ER-regulated gene) reporter line was detectable at 6.25 ng/L at five dpf (Bakos et al., 2019) while GFP induction in *cyp19a1b*-GFP embryos showed an EC_50_ of 1.48 ng/L at four dpf (Hinfray et al., 2016) or 9.87 ng/L at five dpf (exposure from 1-5 dpf) (Petersen et al., 2013). Similarly, Gorelick and Halpern (2011) reported activation of the 5xERE:GFP transgene in the liver and heart at 10 ng/L EE2. Morphological effects have also been observed, among which uninflated swim bladders, pericardial edema, tail malformations, and body axis curvature at 20 ng/L EE2 (Santos et al., 2014). An EC_50_ for gross malformations (e.g., lack of tail formation, scoliosis, yolk sac malformation) was estimated at 57.7 ng/L and reported LC_50_ values ranged from 1.23 to 3.6 mg/L (Baekelandt et al., 2024; Ramirez-Montero et al., 2022). Neurodevelopmental alterations were observed by Vosges et al. (2010), who found a significant increase in GnRH cell bodies in the forebrain and upregulation of AroB expression following exposure to 29.64 ng/L EE2 from 1-5 dpf. In our study, we did not observe any morphological abnormalities up until 50 ng/L (Supplementary Table S3), possibly due to strain differences in sensitivity or lower actual test concentrations than nominal concentrations, as exposure levels were not analytically confirmed. Future studies could investigate potential effects of higher exposure concentrations on HC development.

In contrast, exposure to FUL (an ER antagonist) led to a limited HC increase after continuous exposure to 1 and 2 mg/L (Figure 1D) (in the absence of mortality or malformations, Supplementary Table S3). Namdaran et al. (2012) found impaired HC regeneration after neomycin-induced ablation and 48-h FUL exposure (3–6 mg/L) at 5-6 dpf, but observed no effect in non-ablated FUL-exposed larvae with this shorter exposure window. Hirose et al. (2011) similarly reported no HC changes after 1–6 h exposure to 61 mg/L FUL at five dpf. These discrepancies could reflect differences in test concentrations, exposure duration and developmental timing, with early, continuous exposure potentially influencing outcomes not captured in shorter exposure designs. FUL acts as an ERα/ERβ antagonist and a G-protein-coupled estrogen receptor 1 (GPER) agonist (Boueid et al., 2023). GPER mRNA is expressed in neuromasts of developing zebrafish embryos and in both accessory and hair cells of frogs (Hamilton et al., 2014; Jayasinghe and Volz, 2012). Coty (2024) showed that GPER agonist exposure from five to seven dpf increased HC number in zebrafish. In neuronal systems, GPER activation promotes survival via MAPK, CREB, cAMP, and PI3K pathways (Roque and Baltazar, 2019), which are also critical for HC development and maintenance (Chung et al., 2006; Jadali and Kwan, 2016; Kim et al., 2021; Liu et al., 2021; Wu et al., 2020). Despite this, Coty (2024) observed HC reduction at FUL concentrations similar to those used here. A key distinction lies, again, in exposure timing: Coty initiated treatment at five dpf, whereas our study encompassed LL migration and differentiation from <1 hpf to five dpf. However, it is important to note that only intermediate concentrations resulted in an effect on HCs. Additionally, an effect was only observed in two out of four neuromasts (O1, P1) (Supplementary Figure S4C).

### 4.2 Exposures to methimazole and resorcinol negatively impact lateral line development

Exposure to MMI and RSC, both TPO inhibitors, resulted in a decrease in the number of HCs (Figures 1F,G), but the effect of MMI could not be replicated in a subsequent experiment (Figure 2A, exposure to 300 mg/L MMI from 0 to 120 hpf). This may be attributed to the limited effect size and high variability. Thienpont et al. (2011) reported a LOEC of 34.25 mg/L for impaired T4 synthesis following exposure from 2 to 5 dpf, and Jarque et al. (2018) observed an EC_50_ of 31.85 mg/L for Tg-mCherry fluorescence induction under similar conditions. At those concentrations where decreased numbers of HCs were observed, we also observed impaired swim bladder inflation [a known transient effect of TPO inhibitors, (Stinckens et al., 2018),] and starting from 360 mg/L we also observed impaired pectoral fin development, reduced pigmentation and reduced craniofacial development, all known effects of THSD (OECD, 2023). Similarly, RSC exposures resulting in decreased HC numbers also led to impaired swim bladder inflation and craniofacial development (jaw malformations) (Supplementary Table S3). Van Dingenen et al. (2024) reported decreased whole-body T4 levels from 10.02 mg/L RSC onwards and a 48% incidence of non-inflated swim bladders at 40 mg/L. Taken together, while THSD activity has already been observed at lower concentrations [(Jarque et al., 2018; Thienpont et al., 2011)], the effect on the HCs occurs at concentrations similar to those causing other THSD adverse effects. The behavioral consequences of a reduction in number of HCs remain understudied though existing evidence indicates that, e.g., rheotaxis is altered, but not abolished after chemical insult (Newton et al., 2023). The authors showed that zebrafish with near-complete ablation of HCs still performed rheotaxis although several behavioral metrics were significantly affected. Further research, encompassing a wider range of LL-dependent behaviors is required to determine the impact of the observed effects.

At 1 mg/L IOP (a deiodinase inhibitor) we found a decreased number of HCs, which was not observed at higher exposure concentrations (Figure 1H). We observed impaired swim bladder inflation as of 4 mg/L (45%). Van Dingenen et al. (2023) reported impaired swim bladder inflation as of 1 mg/L IOP (19%) and T4 levels reduced by half at 6 mg/L IOP (no lower concentrations included). Stinckens et al. (2018) reported an EC_50_ of 2.8 mg/L for impaired swim bladder inflation at 168 hpf. Although Gölz et al. (2024a) observed no change in thyroid fluorescence intensity at 2 mg/L, they reported alterations in retinal layers, an endpoint that has also been related to THSD. The highest concentration tested in the present study was 4 mg/L, which lies within the range of reported thyroid-related effects. However, inclusion of higher sublethal concentrations could be necessary to fully capture IOP’s disruptive potential on HC development.

**FIGURE 4:**
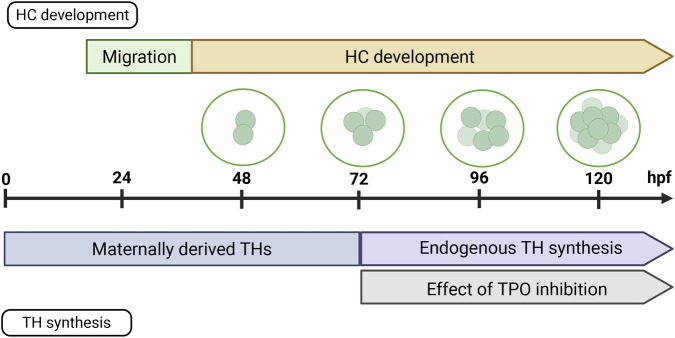
Schematic overview of the embryonal development of hair cells (HCs) and thyroid hormone (TH) system, with a focus on TH synthesis. (Top) HC development: HC migration starts at ± 20 h post fertilization (hpf) and ends at 40 hpf. HC development is a continuous process and they are fully mature 2 weeks post fertilization. (Bottom) TH synthesis: prior to 72 hpf, the embryo is dependent on maternally transferred THs. Afterwards, endogenous TH synthesis becomes active. Thyroperoxidase (TPO) inhibitors, therefore, affect late embryonic development. Developed in B.iorender.

When assessing the impact of THSD on embryonic developmental processes, it is important to consider the parallel timing of development of the TH system on the one hand and the target organ on the other hand. Endogenous TH synthesis in zebrafish is initiated between 60 and 72 hpf (Alt et al., 2006), with early embryonic development relying primarily on maternally transferred THs (Van Dingenen et al., 2023). Embryonic HC migration of the pLL is completed by the onset of endogenous TH synthesis (Figure 4). Since TPO activity is crucial for TH synthesis, TPO inhibition is therefore most likely to impact HC development (rather than migration) during late embryogenesis. A follow-up experiment, using a higher exposure concentration and a shorter exposure window (72–120 hpf) resulted in a reduced number of HCs (Figure 2A). Additionally, the number of neuromasts significantly decreased in the pLL (Figure 2B). The current study cannot definitively exclude a potential effect of TPO inhibition on the number of HCs during early embryonic development (0–72 hpf). This time window was not investigated further (e.g., at higher test concentrations). Higher priority was given to the later time frame when TH synthesis is active. Hu et al. (2019) reported that hypothyroidism in zebrafish leads to a reduced growth rate of trunk neuromasts (pLL) at approximately 7–8 mm standard length (SL), followed by an increase in cranial neuromasts (aLL) around 11 mm SL in the juvenile stage, indicating that TH signaling plays a role in LL system development. This early-stage pattern aligns with our findings as a reduction in number of neuromasts of the pLL was observed, already at 4 mm SL. Hu et al. (2019) proposed that THs regulate the onset of stitch formation, the process by which new neuromasts arise from existing ones (Thomas et al., 2015). Based on these and the current study’s findings, the delayed stitching reported by Hu et al. (2019) may be linked to the reduced HC numbers observed here. Moreover, in zebrafish, interactions between TH receptors (TRs) and retinoic acid (RA) receptors (RARs) are essential for neural crest development (Bohnsack and Kahana, 2013), and RA signaling has been specifically implicated in pLL formation (Nikaido et al., 2017). It should be noted that MMI can cause oxidative stress due to peroxidase inhibition (Amara et al., 2011; Cano-Europa et al., 2011; Cano-Europa et al., 2008; Sefi et al., 2014), and this may have contributed to the observed effect.

The role of THs in HC development and regeneration is well characterized in mammals, whereas comparable data for fish, amphibians, birds, and reptiles remain scarce and poorly understood (Haigis et al., 2023). In mammals, knockdown models targeting TH transporters (Garcia-Aldea et al., 2024; Sharlin et al., 2018), TH receptors (Affortit et al., 2022) or TH conversion (Ng et al., 2004) consistently show that TH deficiency leads to auditory impairments and loss of cochlear HCs. However, such studies have not been conducted in fish. Current hypotheses propose that THs influence neuromast development through pathways such as FGF signaling, Notch signaling, Wnt signaling and BMP signaling, which are critical for LL development and potentially modulated by TH levels. Yet, the precise mechanisms by which THs regulate these pathways in neuromasts remain unclear.

To get more insight into the mechanisms behind HC disruption caused by TPO inhibition, gene transcript level analysis of HC relevant genes was performed (Figure 3). For this analysis, MMI exposure during late embryonic development with active TH synthesis (72–120 hpf) was selected to minimize potential non-specific effects of general peroxidase inhibition. Head (Figure 3A) and body (Figure 3B) samples were analyzed separately to distinguish between aLL and pLL. Transcript levels of *tpo* were elevated after MMI exposure in head samples, probably reflecting a compensatory mechanism and confirming the effect on TPO function. A significant decrease in *otofb* transcript levels was observed. Otofb regulates neurotransmitter release from sensory HCs (Li et al., 2021) and a decrease may reflect a reduced number of active HCs as measured with the FM1-43 dye. Transcript levels of *notch3* and *fgfr1a*, both associated with support cells, were significantly upregulated. *Fgfr1a* serves as a marker for non-sensory LL cells, specifically mantle cells (Steiner et al., 2014), which are thought to contribute to HC regeneration (Lee et al., 2016). This may indicate a compensatory pathway to compensate for HC damage. However, this upregulation was only observed in head samples (aLL) while effects on HC numbers and neuromasts were observed in the pLL (Figure 2; Supplementary Figure S5C). *Fgfr1a* is localized in neuromasts, as well as in the brain and pharyngeal region among others (Larbuisson et al., 2013), which makes it difficult to attribute the observed effect solely to changes in neuromasts. *Notch3* transcripts were upregulated in both head and body samples. Notch signaling must be reinitiated after damage for correct fate determination of newly differentiated cells into HC and support cells (Jiang et al., 2014). Upregulation of both *fgfr1a* and *notch3* suggests that MMI exposure and the resulting damage could ultimately lead to regeneration and fate determination of new HCs.

### 4.3 β-Naphthoflavone did not have an effect on the number of hair cells

The AhR is a hepatic nuclear receptor that regulates genes involved in cell growth, differentiation and development (Kojima et al., 2010). AhR activation can increase liver clearance and reduce TH levels (Haigis et al., 2023). In the present study, exposure to BNF (an AhR agonist) reduced the number of HCs at the two highest test concentrations. However, these concentrations also induced systemic toxicity (Supplementary Table S3). No HC effects were observed at lower concentrations, suggesting that AhR agonism did not specifically impact HC development.

## 5 Conclusion

Existing knowledge suggests that EDCs may interfere with normal neuromast development. This was the first study to explore whether neuromast development is affected by exposure to a selection of model EDCs with different modes of action. Exposures to MMI, RSC (both TH synthesis inhibitors) and FUL (ER antagonist) caused alterations in the number of HCs that could be observed in non-protected 5 day old zebrafish embryos at concentrations comparable to those reported for other effects (e.g., impaired swim bladder inflation). CuSO_4_ and MMI elicited differential effects across neuromast types and parts of the LL system (aLL vs. pLL). This indicates that neuromasts exhibit distinct dose-response profiles, underscoring the need to account for neuromast-specific responses when interpreting LL toxicity. It should be noted that impaired development of the LL, similar to many other effects, is not specific to ED and can also be caused by other mechanisms. Further research, including pharmacological rescue experiments or genetic modulation of TH pathway components, is needed to clarify the causal relationship between ED and HC disruption as well as the underlying mechanisms. Limitations of this study involve high variability in the observed responses. This variation may arise from biological or technical variation, but their relative contribution cannot be determined at present. Future studies employing more sensitive or functionally relevant methods such as histological and whole-mount *in situ* hybridization may offer improved resolution for assessing neuromast development. Moreover, behavioral studies are warranted to assess the functional relevance of the observed effects, given the relatively small magnitude of observed changes.

## Data Availability

The original contributions presented in the study are included in the article/Supplementary Material, further inquiries can be directed to the corresponding author.

## Supplementary material

SUPPLEMENTARY TABLE S1 Abbreviations and full names of the compounds used in this study.

SUPPLEMENTARY DATA SHEET S1 Supplement to materials and methods (Housing fish and egg production, technical details chemicals, preparation of stock solutions, details on positive, negative and solvent controls, assessment of neuromast development, reverse transcription quantitative PCR, data analysis); supplement to results; supplementary references.
