# Supplementary material for: Zebrafish neuromast development: a target for endocrine disrupting chemicals?
Source: Front Toxicol. 2026 Mar 17;8:1733477. doi: 10.3389/ftox.2026.1733477 (PMC13035304; doi:10.3389/ftox.2026.1733477)
Supplement: Supplementary file 1 [file DataSheet1.pdf]

Zebrafish neuromast development: a target for endocrine disrupting chemicals?

Ellen Vandeputte[1], Evelyn Stinckens[1], Jade Verreth[1], Simone Fibiger Sørensen[2], Erik Fransen[3], Henrik Holbech[2], Lucia Vergauwen[1,4], Dries Knapen[1,*]

[1]Zebrafishlab, Veterinary Physiology and Biochemistry, Department of Veterinary Sciences, University of Antwerp, Universiteitsplein 1, 2610 Wilrijk, Belgium.

[2]University of Southern Denmark, Department of Biology, Campusvej, 55, 5230, Odense M, Denmark.

[3]Center of Medical Genetics, University of Antwerp and Antwerp University Hospital, Prins Bouwdewijnlaan 43, 2650 Edegem, Belgium.

[4]ECOSPHERE, Departement of Biology, University of Antwerp, Groenenborgerlaan 171, 2020, Antwerpen, Belgium.

Table of Contents

1 Supplement to materials and methods 2

1.1 Housing fish and egg production 2

1.2 Technical details chemicals 2

1.3 Preparation of stock solutions 2

1.4 Details on positive, negative and solvent controls 2

1.5 Assessment of neuromast development 3

1.6 Reverse transcription quantitative PCR 4

1.7 Data analysis 6

2 Supplement to results 7

3 Supplementary references 20

1 Supplement to materials and methods

1.1 Housing fish and egg production

Wild type broodfish were housed in a ZebTEC stand-alone system (Techniplast, Buguggiate, Italy) and kept in reconstituted fresh water (basis reverse osmosis (RO) water, conductivity 500 ± 50 µS (Instant Ocean® Sea Salt, Blacksburg, USA), pH 7.5 ± 0.3 ($NaHCO3$)), a constant temperature of 28.0 ± 0.2 °C and a 14/10 h light/dark cycle in compliance with EU directive 2010/63/EU. Water quality parameters (ammonia, nitrite and nitrate) were monitored twice a week using Hanna instruments kits (Woonsocket, Rhode Island, USA), with concentrations consistently maintained below 0.1, 0.03, and 12.5 mg/L, respectively. On weekdays, fish were fed twice daily with granulated feed (1.5% of average wet weight; Zebrafeed, Sparos, Olhão, Portugal) and once with prey such as Chironomidae or Chaoboridae larvae, Daphnia sp., or Artemia sp. nauplii (Aquaria Antwerp bvba, Aartselaar, Belgium). On weekends, fish were fed granulated food at least once a day (3% of their mean wet weight, Gemma Micro, Skretting, Stavanger, Norway).

1.2 Technical details chemicals

Table S1: technical details on test chemicals.

Name	CAS	Supplier	Purity	Solvent
Copper sulfate pentahydrate	7758-99-8	VWR chemicals	≥98%	-
17α-Ethinylestradiol	57-63-6	Sigma-Aldrich	≥ 98%	0.01% DMSO
Fulvestrant	129453-61-8	TCI Europe	> 98.0%	0.1% DMSO
β-Napthoflavone	6051-87-2	Acros Organics	>99%	0.1% DMSO
Methimazole	60-56-0	Sigma Aldrich	>99%	-
Resorcinol	108–46–3	Sigma Aldrich	99 %	-
Iopanoic acid	96-83-3	TCI Europe	>98.0%	-

1.3 Preparation of stock solutions

$CuSO_4$ stock solution (0.1 g/L) was prepared in reconstituted fresh water (same as adult housing) containing 1% nitric acid. MMI (15 g/L) and RSC (1 g/L) stock solutions were dissolved in reconstituted fresh water. IOP stock solution (0.1 g/L) was dissolved in reconstituted fresh water containing 0.1M NaOH by sonication (±15 min). Stock solutions of $CuSO_4$, MMI and IOP were stored at room temperature, protected from light. Stock solutions of EE2 (0.5 g/L), FUL (40 g/L) and BNF (1 g/L) were prepared in 100% dimethyl sulfoxide (DMSO, CAS: 67-68-5, Sigma-Aldrich, Saint Louis, USA, ≥99.9% purity). EE2, FUL and BNF stock solutions were stored in the dark (wrapped in aluminum foil) at -20°C. Before use, they were thawed and vortexed.

1.4 Details on positive, negative and solvent controls

A positive control plate containing 4 mg/L 3,4-dichloroaniline (DCA; CAS: 95-76-1, Sigma-Aldrich, Saint Louis, USA) assessed embryo sensitivity. Internal negative control mortality remained below 25%, while positive controls consistently exceeded 30% mortality, validating the assays as per OECD TG 263 (OECD, 2025).

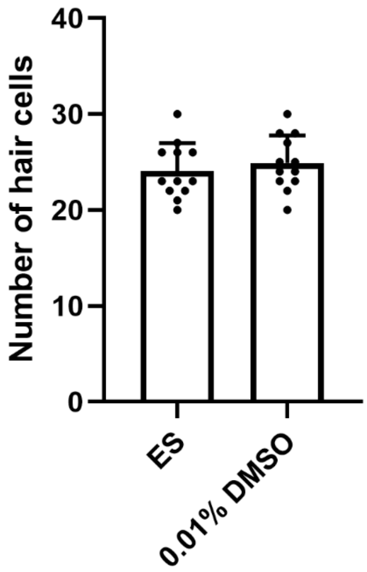

Figure S1: Comparison in number of hair cells between embryo solution (ES, negative control) and 0.01% dimethyl sulfoxide (DMSO). HCs were counted in two neuromasts (O1, P1) and averaged. Data are represented as mean ± standard deviation (SD). n =12.

1.5 Assessment of neuromast development

FM1-43 staining solution was prepared fresh each day by diluting the frozen, light-protected stock in pre-warmed reconstituted freshwater. All steps were performed under low-light conditions (e.g. room lights turned off, window shades remained closed) and the working solution was stored in a brown glass vial in the incubator until use. For staining, embryos were placed in a small mesh filter and submerged in 7.5 mL FM1-43 solution in a 6-well plate. After 45s, the filter with embryos was transferred to reconstituted freshwater and eleutheroembryos were moved to a clean mesh filter in new reconstituted fresh water. Eleutheroembryos underwent 45s washes in fresh reconstituted freshwater for a total of four washes. Each compound and time window experiment was evaluated on two assessment days. After staining, eleutheroembryos were kept in the dark on a heating plate. The coefficient of variation of HC counts in negative controls was 25%, 6%, 8% and 7% for the CuSO4, MMI, RSC and IOP experiments respectively.

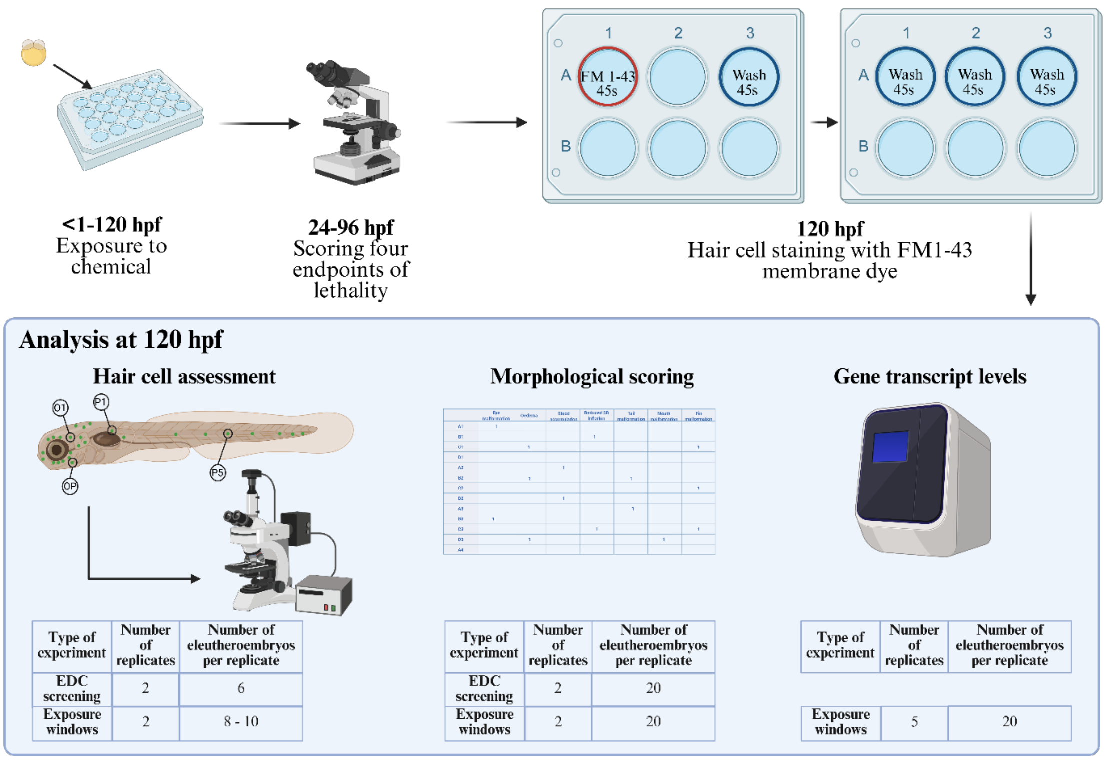

Figure S2: Schematic overview of experimental procedures. Embryos were exposed from <1 hours post fertilization (hpf) until 120 hpf for all experiments. Every 24h, 4 indicators of lethality were observed. At 120 hpf, a full morphological scoring was performed as well as hair cell (HC) staining with FM1-43 membrane dye. At 120 hpf, HCs in four neuromasts were counted, a morphological scoring was performed and samples were obtained for gene transcript analysis. Tables give an overview of the number of replicates and eleutheroembryos analyzed per experiment. Developed in Biorender.

1.6 Reverse transcription quantitative PCR

Separate exposures in polypropylene pots were performed to collect samples, housing 40 embryos in 200 mL medium per pot. Exposures were carried out as described in section 2.2 and 2.3 in the main manuscript. Five independent biological replicates of 20 eleutheroembryos each were sampled at 120 hpf. Eleutheroembryos were euthanized in 1 g/L MS-222 and rinsed three times in ES before being transferred to diethyl pyrocarbonate (DEPC, Sigma-Aldrich, CAS: 1609-47-8) treated water. Microdissection was performed using 5mm forceps (Dumont): a straight cut was made behind the otolith to seperate head (aLL) from abdomen and body (pLL), along with swim bladder and remaining egg yolk. Samples were snap frozen in liquid nitrogen and stored at -80°C until RNA extraction.

RNA was extracted using the Qiagen RNeasy RNA extraction kit (Qiagen, Hilden, Germany) according to manufacturer's instructions. RNA integrity was analyzed using a 12-capillary fragment analyzer (VIB Center for Molecular Neurology, Wilrijk, Belgium) and a DNF-741 RNA kit (15 nt; Agilent). The Thermo Scientific RevertAid H minus First Strand cDNA synthesis Kit (Thermo Fisher Scientific Inc., Waltham, United States) was used for efficient synthesis of first strand cDNA according to manufacturer's instructions. RNA and cDNA concentration as well as purity was measured using a Biodrop µLite+ (Biochrom Ltd, Cambridge, United Kingdom). For RNA, A260/230 and A260/280 ratios were 1.846 to 2.508 and 2.000 and 2.110, respectively. All cDNA A260/230 and A260/280 ratios were 1.730 to 2.038 and 1.714 to 1.770, respectively. Before RT-PCR, cDNA was diluted with RNAse free water to a concentration of 70 ng/µL. RT-qPCR analysis was performed using QuantStudio 3 (ThermoFisher Scientific, Waltham, MA, USA). Wells of a 96-well plate (semi-skirted, FAST type, AmpliStar-II; Westburg) were filled with 5µL (350 ng) cDNA and 15 µl of master mix per reaction. This master mix contained 1 µl of both forward primer (10 µM) and reverse primer (10 µM), 8 µl of DEPC water and 5 µl SYBR green kit, CAPITAL (ROX included) (Biotechrabbit, Berlin, Germany) per reaction. RT-qPCR primers were obtained from Eurogentec (table S2). The thermal cycling program consisted of 95°C for 3 min, 95°C for 20s, annealing temperature for 15s, 72°C for 30s. Melting curves were

analyzed to confirm specific amplification. Primer efficiencies were determined for each run using duplicate standard curves of 5 sequential dilutions of a mixed reference sample. Each cDNA sample was run in two technical replicates which were averaged for transcript analysis. In order to avoid inter-run variation, all samples for a given gene were analyzed in the same run. From a list of reference genes, *actb1* and *rpn2* were chosen as most stable genes using GeNorm. A primer efficiency corrected delta-delta Ct method was used to calculate fold changes, which were normalized using the geometric mean of the two selected reference genes (Pfaffl, 2001, Vandesompele et al., 2002).

Table S2: Primer sequences used in reverse transcription quantitative polymerase chain reaction (RT-qPCR) analysis. Ta = annealing temperature

Group	***Gene***	***Full name***	***Primer sequence (5' --> 3')***	***Ta (°C)***	***Source***
Reference genes	*hprt1*	hypoxanthine guanine phosphoribosyl transferase	F: CAAAATGACCAGTCCACAGG<br>R: TGTCCTCTTCACCAGCAAACT	64	Liu et al. (2011)
	arnt2	aryl-hydrocarbon receptor nuclear translocator 2	F: ACCAACGTGATTGGCTAC<br>R: CCACTCTCGGTTCTTCAT	60	(Van Dingenen et al., 2024)
	actb1	actin, beta1	F: AAGTGCGACGTGGACA<br>R: GTTTAGGTTGGTCGTTCGTTTGA	60	Gonzalez et al. (2006)
	18S	ribosomal 18S	F: CGGAGAGGGAGCCTGAGAA<br>R: AGTCGGGAGTGGGTAATTTGC	60	Biga et al. (2005)
	rpn2	ribophorin II	F: TTGAGTTCAGCCAGCGT<br>R: TGGCAACAAATCGGCG	60	De Wit et al. (2008)
Thyroid hormone system	*tpo*	Thyroid peroxidase	F: TGCCACGGACGAAGAATACC<br>R: CGAACCGGAGGAAGTTGGAA	62	Song et al. (2021)
Hair cell markers	*s100t*	S100 calcium binding protein T	F: TGGGAATGAGGGTGACAAAT<br>R: TCATTCGCTGGTCATGTGTT	60	Barta et al. (2018)
	cabp2b	Calcium binding protein 2b	F: AGCTTCTCTCAGCTCITCAATCTC<br>R: GGCAAAGTGAAACGGGCCTGTC	62	Barta et al. (2018)
Synaptic transmission	*otofb*	Otoferlin b	F: GCAAAGACGGCAAAGCAGTGC<br>R: GCTTCCACTTTGCCTGTAATTTCTAGCTCATC	64	Chatterjee et al. (2015)
	rims2	regulating synatpic membrane exocytosis2	F: TTACCAGCAGCAGCTGTCATTCG<br>R: AAGAACTCGACGTCTCGGTTCTC	60	McDermott et al. (2007)
	synj1	synaptojanin 1	F: GACATCCAACCAAGACCCAACAG<br>R: ACAGGTCATTATATCTGCACTTTTATA	60	McDermott et al. (2007)
Support cells	*Notch3*	Notch receptor 3	F: TAAGTCCAGTAAGCGTGCCG<br>R: AGCAATGGGAGGCAGTTTGA	64	Sahu et al. (2021)
	fgfr1a	fibroblast growth factor receptor 1a	F: CTGCCATATGTCCGAGCCTT<br>R: GTGATGGGAGTGGCCGATAG	62	Steiner et al. (2014)
Hair cell integrity	*myo6b*	myosin VIb	F: TTGCGCAGAGATGCTACCAC<br>R: CAGCTCAGCGTACTTCCACT	68	Li et al. (2021)

1.7 Data analysis

All statistical analyses were performed in R (v 4.5.0) unless otherwise stated, with statistical significance defined as $p < 0.05$. Morphological malformations and mortality were analyzed using a binary logistic regression (Michiels et al., 2017). An odds ratio was calculated in case of a 'zero cell count' (when all eleutheroembryos in a group have the same score, e.g. 100 % oedema) by adding a small value (0.5) to each cell (Pype et al., 2015). For the analysis of HC counts, separate statistical models were run per compound. The effect of treatment on number of HCs was tested with a linear regression model with the sum of all HC counts within one eleutheroembryo as dependent variable. Treatment was entered as categorical independent variable, and the replicate (assessment day) was added as covariate to account for a possible batch effect (regardless of its significance). Pairwise comparison between treatments was performed using an mvt correction for multiple hypothesis testing, using the control as reference. The effect of the treatment on the neuromast-specific HC count was studied using a linear mixed model, with HC count as dependent variable. Fixed effects included neuromast, treatment and their interaction, adding the replicate as covariate. Embryo ID was added as random effect to account for the dependence between observations within the same embryo. The significance of the interaction term showed whether the effect of the treatment was different between neuromasts. The effect of treatment on HC count within the separate neuromasts was modeled using a linear regression model, with treatment as independent variable and replicate as covariate, and a pairwise comparison between treatments with a mvt correction as described above. Across all fitted models, effect sizes and confidence intervals for the pairwise comparisons were calculated using the emmeans and multcomp packages in R. The assumptions for linear mixed models and linear regression (normality of residuals and homoskedasticity) were visually inspected. Gene transcript level analysis was performed in GraphPad (GraphPad Software, version 10.0.2, San Diego, CA). Data was tested for normality, followed by either an unpaired t-test or Mann-Whitney U test.

2 Supplement to results

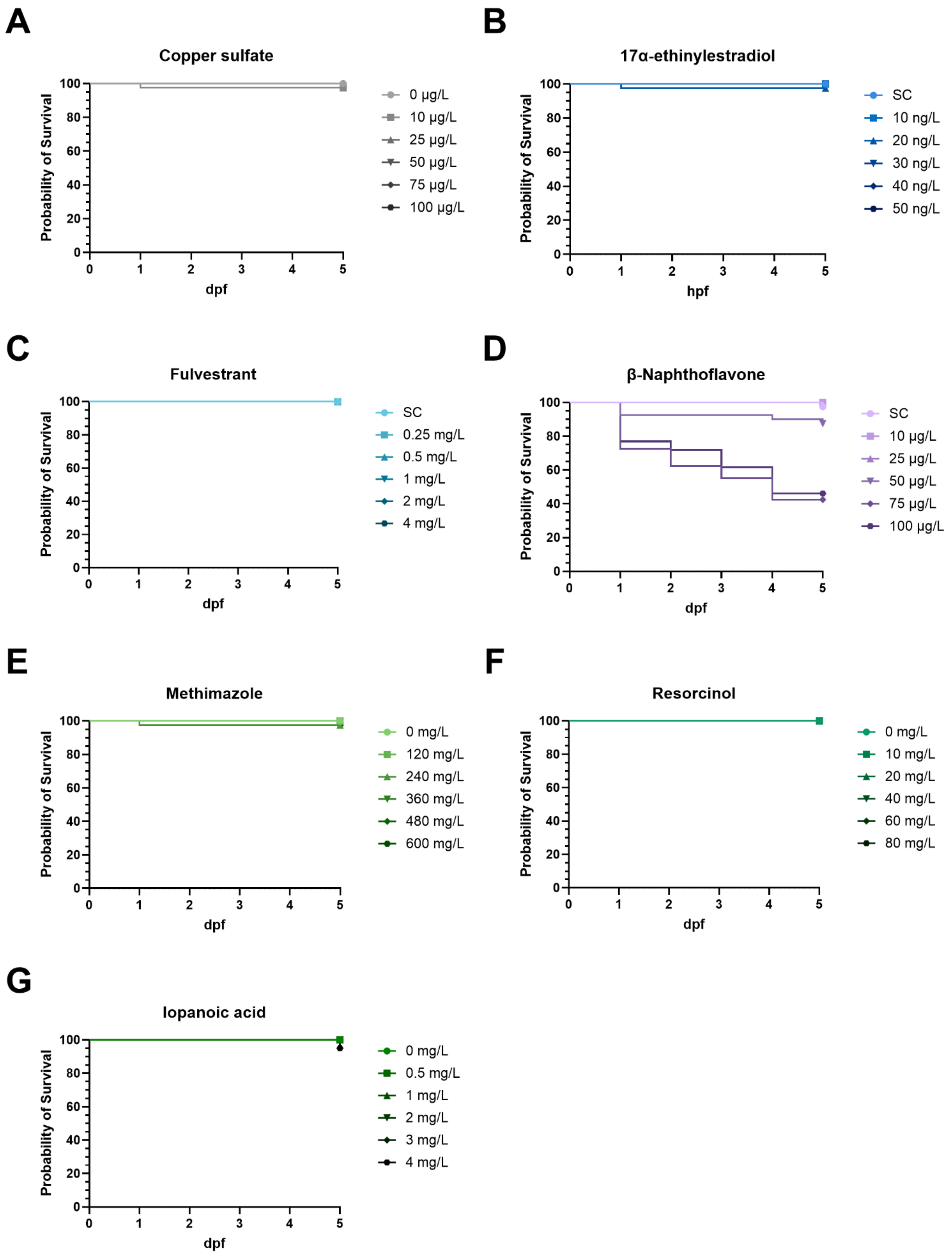

Figure S3: Probability of survival. **(A)** Copper sulfate, **(B)** 17-α-ethynilestradiol, **(C)** Fulvestrant, **(D)** β-napthoflavone, **(E)** methimazole, **(F)** resorcinol, **(G)** iopanoic acid . Dpf: days post fertilization.

Table S3: Overview of the effects on morphology after exposure until 120 hours post fertilization (hpf) to copper sulfate pentahydrate ($CuSO_4$), 17α-ethinylestradiol (EE2), fulvestrant (FUL), β-napthoflavone (BNF), methimazole (MMI), resorcinol (RSC) and iopanoic acid (IOP). Results are shown as %, except for larval length (mm). Sample size (n) = 40 for morphological scorings, n = 24 for larval length analysis. Mortality was assessed daily throughout embryonic development (24-120 hpf) and a delay in hatching was observed at 72 hpf. All other endpoints were assessed at 120 hpf. Statistical difference from controls ($p < 0.05$) marked in bold with *.

Chemical	Concentration	Mortality	Delay in hatching	Curvature spine	Oedema	Blood accumulation	Malformation pectoral fin	Malformation cardiovascular system	Malformation head	Deviating pigment	Uninflated swim bladder	Larval length
CuSO4 (µg/L)	0	-	-	-	-	-	-	-	-	-	7.5	4.02
	10	2.5	-	-	-	-	-	-	-	-	5.3	3.92
	25	-	2.5	2.5	-	-	-	-	-	-	7.5	3.96
	50	2.5	18	-	-	-	-	-	-	-	**67.5***	**3.80***
	75	-	**52.5***	-	-	-	-	-	-	-	**100***	**3.78***
	100	-	**75***	2.5	2.5	-	-	-	-	-	**100***	**3.69***
EE2 (ng/L)	0	-	-	-	-	-	-	-	-	-	7.5	3.87
	10	-	-	-	-	-	-	-	-	-	-	3.96
	20	2.5	-	5.1	-	-	-	-	-	-	5	3.98
	30	-	-	-	-	-	-	-	-	-	-	3.99
	40	-	-	-	-	-	-	-	-	-	-	**4.04***
	50	-	-	-	-	2.5	-	-	-	-	-	3.94
FUL (mg/L)	0	-	-	-	-	-	-	7.5	-	-	-	4.02
	0.25	-	-	-	-	-	-	5	-	-	-	3.97
	0.5	-	-	-	-	2.5	-	5	-	-	-	4.00
	1	-	-	-	2.5	-	-	5	-	-	-	4.00
	2	-	-	-	2.5	-	-	7.5	2.5	-	2.5	4.01
	4	-	-	-	-	-	-	10	-	-	-	4.01
BNF (µg/L)	0	2.5	-	-	-	-	-	2.6	-	-	-	4.01
	10	-	-	-	-	-	-	7.5	5	-	5	3.97
	25	-	-	-	-	-	-	7.5	2.5	-	12.5	3.97
	50	12.5	-	3.1	5.8	8.4	-	**33.6***	11.5	-	**52.8***	**3.81***
	75	**57.5***	10	12.1	34.3	36.4	-	**75.7***	**67.9***	-	**95***	-
	100	**52.5***	12.5	9.1	34.9	38.6	4.6	**69.8***	**56.8***	-	**95.8***	**3.81***
MMI (mg/L)	0	-	-	-	-	2.5	-	-	-		2.5	3.93
	120	-	-	-	-	-	-	-	-	-	7.5	3.96
	240	2.5	12.8	2.5	-	5.1	-	-	2.6	-	**38.7**	3.87

	360	-	**100***	5	-	2.5	**40***	2.5	**82.5***	**40***	**92.5***	3.87
	480	-	**100***	12.5	-	5	**45***	2.5	**97.5***	**90***	**100***	3.83
	600	2.5	**100***	**59.1***	15.5	2.6	**46.3***	2.5	**100***	**100***	**100***	**3.71***
RSC (mg/L)	0	-	-	-	-	-	-	-	-	-	-	3.94
	10	-	-	2.5	2.5	-	-	-	-	-	7.5	3.96
	20	-	-	-	-	-	-	-	-	-	2.5	3.97
	40	-	-	-	-	-	-	-	7.5	-	**87.5***	3.83
	60	-	-	2.5	-	-	-	-	**42.5***	-	**97.5***	3.97
	80	-	-	-	-	-	-	-	**85***	-	**100***	3.96
IOP (mg/L)	0	-	-	2.5	2.5	-	-	2.5	2.5	-	7.5	4.05
	0.5	-	-	-	-	-	-	-	-	-	5	4.02
	1	-	-	-	2.5	-	-	-	-	-	2.5	3.99
	2	-	-	-	-	2.5	-	-	-	-	7.5	3.97
	3	-	-	2.5	-	-	-	-	2.5	-	22.5	4.00
	4	5	-	8.1	10.8	2.8	-	10.8	10.8	-	**45***	4.01

Table S4: Overview of the impact of time window exposures with methimazole (MMI) on morphological malformations. Mortality was assessed daily throughout embryonic development (24-120 hpf). All other endpoints were assessed at 120 hpf. Results are shown as % and sample size = 40. Statistical difference from controls ($p < 0.05$) marked in bold with *.

Time window (hpf)	Concentration (mg/L)	Mortality	Curvature spine	Oedema	Blood accumulation	Malformation pectoral fin	Malformation cardiovascular system	Malformation head	Deviating pigment	Uninflated swim bladder	Larval length
0-120	NC	-	5	-	-	-	-	-	-	2.5	3.93
0-72	300	-	5	-	-	-	-	2.5	-	17.5	**4.01***
72-120	300	-	10	-	-	-	-	-	-	15	3.96
0-120	300	-	2.5	-	-	-	-	5	-	**67.5***	3.92
0-120	NC	-	-	-	-	-	-	-	-	10	3.87
72-120	400	-	7.5	-	-	-	-	-	-	12.5	3.84
72-120	500	-	5	-	-	-	-	-	-	**32.5***	3.79

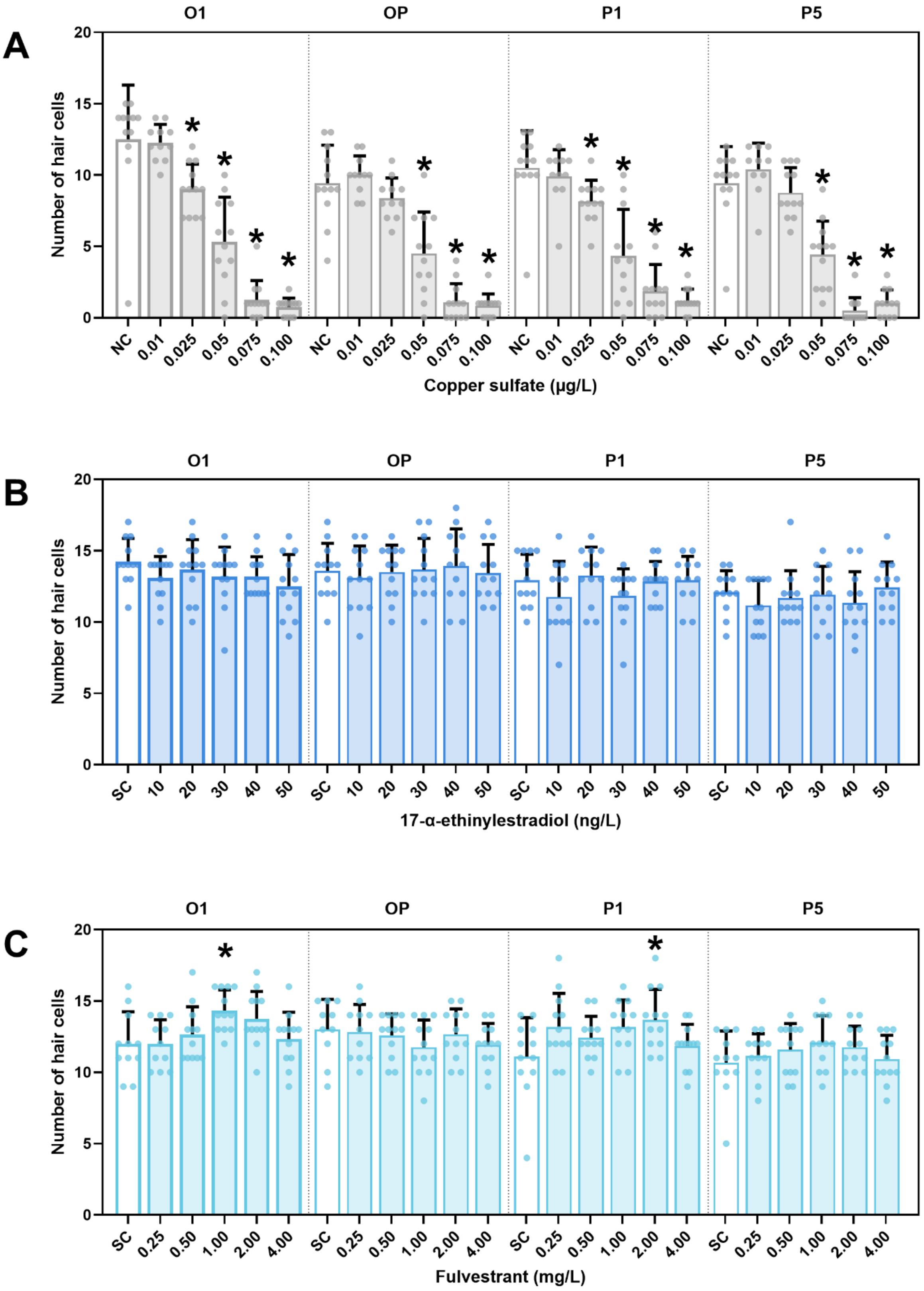
A
O1
OP
P1
P5
Number of hair cells
NC 0.01 0.025 0.05 0.075 0.100
Copper sulfate (µg/L)
B
SC 10 20 30 40 50
17-α-ethinylestradiol (ng/L)
C
SC 0.25 0.50 1.00 2.00 4.00
Fulvestrant (mg/L)

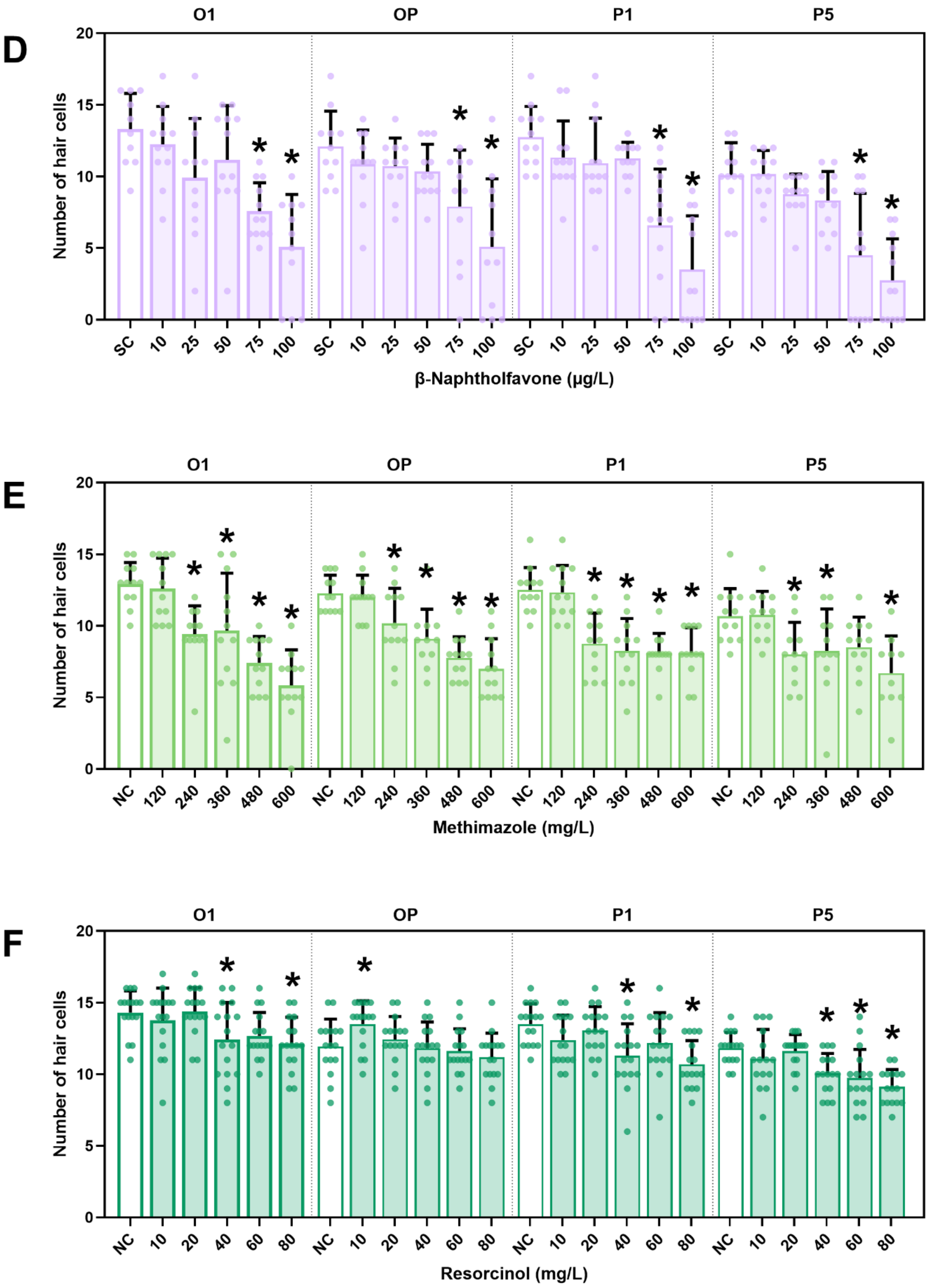
D
O1
OP
P1
P5
Number of hair cells
SC
10
25
50
75
100
β-Naphtholfavone (µg/L)
E
Number of hair cells
NC
120
240
360
480
600
Methimazole (mg/L)
F
Number of hair cells
NC
10
20
40
60
80
Resorcinol (mg/L)

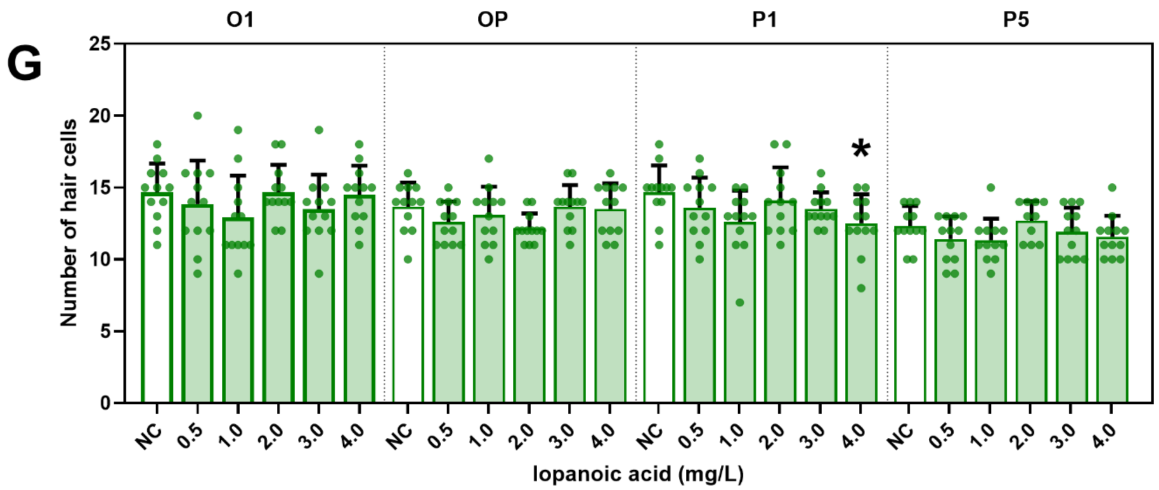

Figure S4: Initial screening of endocrine disrupting compounds and positive control on the number of hair cells (HCs) in four neuromasts (O1, OP, P1 and P5) in 120 hours post fertilization (hpf) zebrafish eleutheroembryos. **(A)** Copper sulfate, **(B)** 17-α-ethynilestradiol, **(C)** Fulvestrant, **(D)** β-naphthoflavone, **(E)** methimazole, **(F)** resorcinol, **(G)** iopanoic acid. Figure 1 in the main manuscript shows the averages across these 4 neuromasts. Data are represented as mean ± standard deviation. Sample size (n): 12 eleutheroembryos per concentration, except for BNF (n= 11 in higher concentrations, due to mortality). Statistical difference from controls: $p < 0.05$.

Table S5: Results of endocrine disrupting compound (EDC) exposure on the number of hair cells (HCs): copper sulfate pentahydrate ($CuSO_4$), 17α-ethinylestradiol (EE2), fulvestrant (FUL), β-napthoflavone (BNF), methimazole (MMI), resorcinol (RSC) and iopanoic acid (IOP). Effect size, confidence interval (CI) and p-value are given. Statistical difference from controls ($p < 0.05$) marked in bold with *.

Chemical	Type of analysis	Comparison	Effect size	CI (lower, upper)	p-value
CuSO4 (µg/L)	Sum of HCs	0-10	1.492	(-6.41, 9.39)	0.9850
		0-25	-7.413	(-15.11, 0.28)	0.0628
		0-50	-23.250	(-30.77, -15.73)	**<0.001***
		0-75	-37.167	(-44.69, -29.65)	**<0.001***
		0-100	-38.083	(-45.61, -30.56)	**<0.001***
	NM_O1	0-10	-0.2128	(-2.69, 2.27)	0.9999
		0-25	-3.5000	(-5.92, -1.08)	**0.00194***
		0-50	-7.1667	(-9.59, -4.74)	**< 0.001***
		0-75	-11.2500	(-13.67, -8.83)	**< 0.001***
		0-100	-11.7500	(-14.17, -9.33)	**< 0.001***
	NM_OP	0-10	0.5708	(-1.51, 2.66)	0.931
		0-25	-1.0656	(-3.15, 1.02)	0.553
		0-50	-4.9167	(-6.95, -2.88)	**< 0.001***
		0-75	-8.3333	(-10.37, -6.30)	**< 0.001***
		0-100	-8.5833	(-10.62, -6.55)	**< 0.001***
	NM_P1	0-10	-0.5667	(-2.87, 1.74)	0.9359
		0-25	-2.333	(-4.58, -0.08)	**0.0397***
		0-50	-6.1667	(-8.42, -3.92)	**< 0.001***
		0-75	-8.6667	(-10.92, -6.42)	**< 0.001***
		0-100	-9.3333	(-11.58, -7.08)	**< 0.001***
	NM_P5	0-10	1.023	(-1.02, 3.07)	0.573
		0-25	-0.6667	(-2.61, 1.28)	0.848
		0-50	-5.0000	(-6.94, -3.05)	**<1e-04***
		0-75	-8.9167	(-10.86, -6.97)	**<1e-04***
		0-100	-8.4167	(-10.36, -6.47)	**<1e-04***
EE2 (ng/L)	Sum of HCs	SC-10	-3.4170	(-8.24, 1.40)	0.249
		SC-20	-0.7500	(-5.57, 4.07)	0.994
		SC-30	-2.2500	(-7.07, 2.57)	0.633
		SC-40	-1.5830	(-6.40, 3.23)	0.866
		SC-50	-1.5830	(-6.40, 3.23)	0.866
	NM_O1	SC-10	-1.1667	(-3.05, 0.72)	0.369
		SC-20	-0.5833	(-2.47, 1.30)	0.890
		SC-30	-1.0833	(-2.97, 0.80)	0.439
		SC-40	-1.0833	(-2.97, 0.80)	0.439
		SC-50	-1.7500	(-3.63, 0.13)	0.077
	NM_OP	SC-10	-0.5000	(2.78, 1.78)	0.971
		SC-20	-0.0833	(-2.36, 2.19)	1.000
		SC-30	-0.0833	(-2.19, 2.36)	1.000
		SC-40	-0.3333	(-1.94, 2.61)	0.995
		SC-50	-0.1667	(-2.44, 2.11)	1.000
	NM_P1	SC-10	-1.1670	(-3.01, 0.073)	0.378
		SC-20	-0.3333	(-1.57, 2.23)	0.989

		SC-30	-1.0830	(-2.98, 0.82)	0.448
		SC-40	-0.0833	(-1.98, 1.82)	1.000
		SC-50	-2.949e-15	(-1.90, 1.90)	1.000
	NM_P5	SC-10	-0.9167	(-2.82, 0.99)	0.606
		SC-20	-0.4167	(-2.32, 1.49)	0.971
		SC-30	-0.1667	(-2.07, 1.74)	1.000
		SC-40	-0.7500	(-2.65, 1.15)	0.764
		SC-50	-0.3333	(-1.57, 2.24)	0.989
FUL (mg/L)	Sum of HCs	SC-0.25	3.053	(-2.04, 8.15)	0.400
		SC-0.50	4.553	(-0.40, 9.56)	0.081
		SC-1.00	6.667	(1.69, 11.65)	**0.0044***
		SC-2.00	7.167	(219, 12.15)	**0.0020***
		SC-4.00	2.333	(-2.65, 7.31)	0.6198
	NM_O1	SC-0.25	0.0859	(-1.90, 2.07)	1.0000
		SC-0.50	0.7069	(-1.23, 2.65)	0.8049
		SC-1.00	2.3763	(0.44, 4.31)	**0.0109***
		SC-2.00	1.7930	(-0.14, 3.73)	0.0786
		SC-4.00	0.3763	(-1.56, 2.31)	0.9818
	NM_OP	SC-0.25	1.1190	(-1.11, 3.35)	0.569
		SC-0.50	0.8333	(-1.35, 3.01)	0.785
		SC-1.00	-4.293e-15	(-2.18, 2.17)	1.000
		SC-2.00	0.9167	(-1.26, 3.10)	0.718
		SC-4.00	0.1667	(-2.01, 2.35)	1.000
	NM_P1	SC-0.25	2.0833	(-0.11, 4.27)	0.0684
		SC-0.50	1.3333	(-0.86, 3.53)	0.3872
		SC-1.00	2.0833	(-0.11, 4.28)	0.0682
		SC-2.00	2.5833	(0.39, 4.78)	**0.0151***
		SC-4.00	0.7500	(-1.44, 2.94)	0.8471
	NM_P5	SC-0.25	-0.0128	(-1.79, 1.77)	1.000
		SC-0.50	0.4038	(-1.38, 2.19)	0.965
		SC-1.00	0.9039	(-0.88, 2.69)	0.552
		SC-2.00	0.5705	(-1.21, 2.35)	0.873
		SC-4.00	-0.2628	(-2.05, 1.52)	0.995
BNF (µg/L)	Sum of HCs	SC-10	-3.338	(-13.87, 7.12)	0.8717
		SC-25	-9.3738	(-19.87, 1.13)	0.0952
		SC-50	-6.8738	(-17.37, 3.63)	0.3147
		SC-75	-22.0405	(-32.54, -11.54)	**<0.001***
		SC-100	-31.0052	(-41.74, -20.27)	**<0.001***
	NM_O1	SC-10	-1.0500	(-4.58, 2.48)	0.8986
		SC-25	-3.3603	(-6.97, 0.25)	0.0758
		SC-50	-2.1333	(-5.67, 1.40)	0.3848
		SC-75	-5.7167	(-9.25, -2.18)	**<0.001***
		SC-100	-8.2397	(-11.85, -4.63)	**<0.001***
	NM_OP	SC-10	-1.2952	(-4.59, 2.00)	0.7625
		SC-25	-1.3636	(-4.73, 2.00)	0.7413
		SC-50	-1.7952	(-5.09, 1.50)	0.4883
		SC-75	-4.2571	(-7.62, -0.89)	**0.0083***
		SC-100	-7.0000	(-10.36, -3.64)	**<0.001***
	NM_P1	SC-10	-1.4167	(-4.53, 1.70)	0.654

		SC-25	-1.8333	(-4.95, 1.28)	0.416
		SC-50	-1.5000	(-4.61, 1.61)	0.605
		SC-75	-6.1667	(-9.28, -3.05)	**<0.001***
		SC-100	-9.2500	(-12.36, -6.14)	**<0.001***
	NM_P5	SC-10	0.0833	(-2.69, 2.85)	1.000
		SC-25	-1.3333	(-4.10, 1.44)	0.606
		SC-50	-1.7500	(-4.52, 1.02)	0.351
		SC-75	-5.5833	(-8.35, -2.81)	**<0.001***
		SC-100	-7.3333	(-10.10, -4.56)	**<0.001***
MMI (mg/L)	Sum of HCs	0-120	-0.6667	(-6.91, 5.57)	0.999
		0-240	-12.5076	(-18.88, -6.12)	**<1e-04***
		0-360	-11.5076	(-17.88, -5.13)	**<1e-04***
		0-480	-16.5833	(-22.82, -10.35)	**<1e-04***
		0-600	-20.6333	(-27.18, -14.09)	**<1e-04***
	NM_O1	0-120	-0.3333	(-2.83, 2.16)	0.9968
		0-240	-3.5000	(-5.99, -101)	**0.0027***
		0-360	-3.2500	(-5.74, -0.76)	**0.0059***
		0-480	-5.5000	(-7.99, -3.01)	**<0.001***
		0-600	-7.0833	(-9.58, -4.59)	**<0.001***
	NM_OP	0-120	-0.2500	(-2.17, 1.67)	0.997
		0-240	-2.0833	(-4.00, -0.16)	**0.029***
		0-360	-3.1962	(-5.16, -1.23)	**<0.001***
		0-480	-4.5000	(-6.42, -2.58)	**<0.001***
		0-600	-5.2500	(-7.17, -3.33)	**<0.001***
	NM_P1	0-120	-0.1667	(-2.08, 1.75)	1.000
		0-240	-3.7500	(-5.67, -1.83)	**1.7e-05***
		0-360	-4.2500	(-6.17, -2.33)	**< 1e-05***
		0-480	-4.4167	(-6.33, -2.50)	**< 1e-05***
		0-600	-4.4167	(-6.33, -2.50)	**< 1e-05***
	NM_P5	0-120	0.0833	(-2.39, 2.41)	1.000
		0-240	-2.7208	(-5.10, -0.35)	**0.019***
		0-360	-2.4167	(-4.74, -0.09)	**0.038***
		0-480	-2.1667	(-4.49, 0.15)	0.075
		0-600	-3.9667	(-6.40, -1.53)	**<0.001***
RSC (mg/L)	Sum of HCs	0-10	-0.2837	(-4.42, 3.85)	1.000
		0-20	-0.0625	(-4.13, 4.01)	1.000
		0-40	-5.9375	(-10.01, -1.87)	**0.002***
		0-60	-5.3125	(-9.38, -1.24)	**0.006***
		0-80	-8.4375	(-12.51 -4.37)	**<0.001***
	NM_O1	0-10	-0.5625	(-2.32, 1.20)	0.881
		0-20	0.0625	(-1.70, 1.82)	1.000
		0-40	-1.8750	(-3.64, -0.11)	**0.0328***
		0-60	-1.6250	(-3.39, 0.14)	0.081
		0-80	-2.1875	(-3.95, -0.43)	**0.009***
	NM_OP	0-10	1.5625	(0.01, 3.11)	**0.048***
		0-20	0.5000	(-1.05, 2.05)	0.877
		0-40	-0.1250	(-1.67, 1.42)	0.999
		0-60	-0.3125	(-1.86, 1.23)	0.980
		0-80	-0.7500	(-2.30, 0.80)	0.607

	NM_P1	0-10	-1.1250	(-2.75, 0.50)	0.274
		0-20	-0.4375	(-2.07, 1.19)	0.937
		0-40	-2.1875	(-3.82, -0.56)	**0.004***
		0-60	-1.3125	(-2.94, 0.32)	0.155
		0-80	-2.8125	(-4.44, -1.18)	**<0.001***
	NM_P5	0-10	-0.7746	(-2.12, 0.57)	0.447
		0-20	-0.1875	(-1.51, 1.13)	0.996
		0-40	-1.7500	(-3.08, -0.42)	**0.005***
		0-60	-2.0625	(-3.39, -0.74)	**<0.001***
		0-80	-2.6875	(-4.01, -1.36)	**<0.001***
IOP (mg/L)	Sum of HCs	0-0.5	-3.9167	(-8.60, 0.77)	0.131
		0-1.0	-5.4167	(-10.10, -0.73)	**0.018***
		0-2.0	-1.4138	(-6.34, 3.51)	0.917
		0-3.0	-2.7500	(-7.43, 1.93)	0.422
		0-4.0	-3.2500	(-7.93, 1.43)	0.268
	NM_O1	0-0.5	-0.8333	(-3.32, 1.65)	0.856
		0-1.0	-1.7500	(-4.23, 0.73)	0.254
		0-2.0	-6.661e-16	(-2.48, 2.48)	1.000
		0-3.0	-1.1670	(-3.65, 1.32)	0.627
		0-4.0	-1.6670	(-2.65, 2.32)	1.000
	NM_OP	0-0.5	-1.0830	(-2.77, 0.60)	0.335
		0-1.0	-0.5833	(-2.70, 1.10)	0.841
		0-2.0	-1.5000	(-3.17, 0.19)	0.097
		0-3.0	-1.982e-15	(-1.69, 1.69)	1.000
		0-4.0	-0.1667	(-1.85, 1.52)	0.999
	NM_P1	0-0.5	-1.0833	(-3.18, 1.01)	0.541
		0-1.0	-2.0833	(-4.18, 0.01)	0.052
		0-2.0	-0.5833	(-2.68, 1.51)	0.925
		0-3.0	-1.1667	(-3.26, 0.92)	0.470
		0-4.0	-2.1667	(-4.26, -0.07)	**0.040***
	NM_P5	0-0.5	-0.9167	(-2.49, 0.65)	0.426
		0-1.0	-1.000	(-2.57, 0.57)	0.344
		0-2.0	0.4052	(-1.24, 2.05)	0.954
		0-3.0	-0.4167	(-1.99, 1.15)	0.938
		0-4.0	-0.7500	(-2.32, 0.82)	0.614

Table S6: Results of effect of MMI during different time windows on the number of hair cells (HCs): effect size, confidence interval (CI) and p-value. Statistical difference from controls ($p < 0.05$) marked in bold with *.

Concentration (mg/L)	Type of analysis	Time windows (hpf)	Effect size	CI (lower, upper)	p-value
300	Sum of HCs	0-72	-2.9000	(-8.11, 2.31)	0.406
		72-120	-4.6229	(-9.90, 0.65)	0.099
		0-120	-3.5176	(-8.79, 1.76)	0.265
	O1	0-72	-1.0000	(-3.49, 1.49)	0.648
		72-120	-2.0908	(-4.61, 0.43)	0.074
		0-120	-2.5118	(-5.03, 0.01)	**0.042***
	OP	0-72	-0.6000	(-2.19, 0.99)	0.691
		72-120	-1.3809	(-2.99, 0.23)	0.131
		0-120	-1.1704	(-2.78, 0.44)	0.201
	P1	0-72	-0.9000	(-2.84, 1.04)	0.552
		72-120	-0.6472	(-2.61, 1.32)	0.752
		0-120	-0.5420	(-2.51, 1.42)	0.722
	P5	0-72	-0.4000	(-2.02, 1.22)	0.879
		72-120	-0.5039	(-2.14, 1.14)	0.793
		0-120	0.7066	(-0.93, 2.35)	0.642

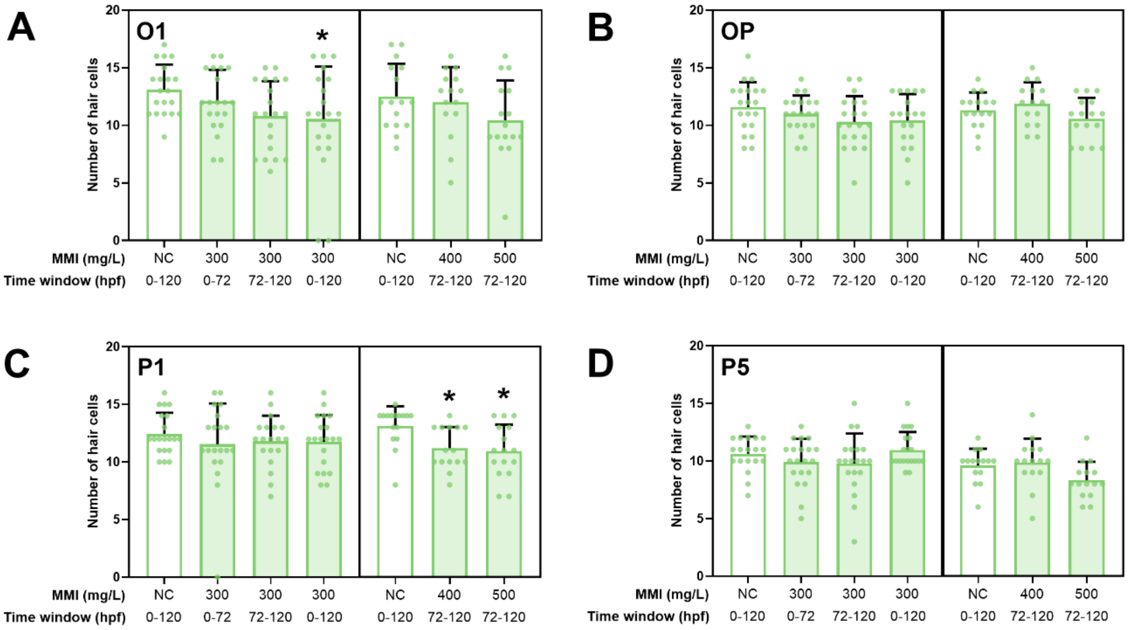

Figure S5: Effect of MMI exposure during different time windows on the total number of hair cells (HCs) at 120 hours post fertilization (hpf). Three different time windows were examined: 0-72 hpf, 72-120 hpf and 0-120 hpf. **(A)** neuromast O1, **(B)** neuromast OP, **(C)** neuromast P1, **(D)** neuromast P5. Figure 2 in the main manuscript shows the averages of these 4 neuromasts. Data are represented as mean ± standard deviation (SD). Sample size = 16-20. Statistical difference ($p < 0.05$) between negative control (NC) and test concentration is denoted with *.

Table S7: **:** Results of effect of MMI during one time window, 72-120 hours post fertilization (hpf), on the number of hair cells (HCs): effect size, confidence interval (CI) and p-value. Statistical difference from controls ($p < 0.05$) marked in bold with *.

Time window (hpf)	Type of analysis	Concentration MMI (mg/L)	Effect size	CI (lower, upper)	p-value
72-120	Sum of HCs	400	-1.4433	(-5.28, 2.39)	0.599
		500	-5.7333	(-9.56, -1.91)	**0.003***
	O1	400	-0.3897	(-3.04, 2.26)	0.921
		500	-1.7333	(-4.38, 0.91)	0.241
	OP	400	0.5052	(-0.97, 1.98)	0.652
		500	-0.8667	(-2.34, 0.61)	0.310
	P1	400	-1.8579	(3.47, -0.25)	**0.022***
		500	-1.8667	(-3.47, -0.26)	**0.021***
	P5	400	0.2992	(-1.15, 1.74)	0.8506
		500	-1.2667	(-2.71, 0.18)	0.0917

Table S8: Results of effect of MMI during different time windows on the number neuromasts in the anterior lateral line (aLL) and posterior lateral line (pLL): effect size, confidence interval (CI) and p-value. Statistical difference from controls ($p < 0.05$) marked in bold with *.

Part of the LL	Time window (hpf)	Concentration (mg/L)	Effect size	CI (lower, upper)	p-value
aLL	72-120	400	-0.1807	(-1.13, 0.77)	0.873
	72-120	500	-0.2998	(-1.24, 0.64)	0.688
pLL	72-120	400	-0.6266	(-2.08, 0.83)	0.517
	72-120	500	-1.6932	(-3.15, -0.24)	**0.020***

3 Supplementary references

BARTA, C. L., LIU, H., CHEN, L., GIFFEN, K. P., LI, Y., KRAMER, K. L., BEISEL, K. W. & HE, D. Z. 2018. RNA-seq transcriptomic analysis of adult zebrafish inner ear hair cells. *Sci Data,* 5**,** 180005.

BIGA, P. R., ROBERTS, S. B., ILIEV, D. B., MCCAULEY, L. A., MOON, J. S., COLLODI, P. & GOETZ, F. W. 2005. The isolation, characterization, and expression of a novel GDF11 gene and a second myostatin form in zebrafish, Danio rerio. *Comp Biochem Physiol B Biochem Mol Biol,* 141**,** 218-30.

CHATTERJEE, P., PADMANARAYANA, M., ABDULLAH, N., HOLMAN, C. L., LADU, J., TANGUAY, R. L. & JOHNSON, C. P. 2015. Otoferlin deficiency in zebrafish results in defects in balance and hearing: rescue of the balance and hearing phenotype with full-length and truncated forms of mouse otoferlin. *Mol Cell Biol,* 35**,** 1043-54.

DE WIT, M., KEIL, D., REMMERIE, N., VAN DER VEN, K., VAN DEN BRANDHOF, E. J., KNAPEN, D., WITTERS, E. & DE COEN, W. 2008. Molecular targets of TBBPA in zebrafish analysed through integration of genomic and proteomic approaches. *Chemosphere,* 74**,** 96-105.

GONZALEZ, P., BAUDRIMONT, M., BOUDOU, A. & BOURDINEAUD, J. P. 2006. Comparative effects of direct cadmium contamination on gene expression in gills, liver, skeletal muscles and brain of the zebrafish (Danio rerio). *Biometals,* 19**,** 225-35.

LI, X. D., TU, H. W., HU, K. Q., LIU, Y. G., MAO, L. N., WANG, F. Y., QU, H. Y. & CHEN, Q. 2021. Effects of Toluene on the Development of the Inner Ear and Lateral Line Sensory System of Zebrafish. *Biomed Environ Sci,* 34**,** 110-118.

LIU, Y., WANG, J., FANG, X., ZHANG, H. & DAI, J. 2011. The thyroid-disrupting effects of long-term perfluorononanoate exposure on zebrafish (Danio rerio). *Ecotoxicology,* 20**,** 47-55.

MCDERMOTT, B. M., JR., BAUCOM, J. M. & HUDSPETH, A. J. 2007. Analysis and functional evaluation of the hair-cell transcriptome. *Proc Natl Acad Sci U S A,* 104**,** 11820-5.

MICHIELS, E. D., VERGAUWEN, L., HAGENAARS, A., FRANSEN, E., DONGEN, S. V., VAN CRUCHTEN, S. J., BERVOETS, L. & KNAPEN, D. 2017. Evaluating Complex Mixtures in the Zebrafish Embryo by Reconstituting Field Water Samples: A Metal Pollution Case Study. *Int J Mol Sci,* 18.

OECD 2025. Test No. 236: Fish Embryo Acute Toxicity (FET) Test. *OECD Guidelines for the Testing of Chemicals.* OECD Publishing.

PFAFFL, M. W. 2001. A new mathematical model for relative quantification in real-time RT-PCR. *Nucleic Acids Res,* 29**,** e45.

PYPE, C., VERBUEKEN, E., SAAD, M. A., CASTELEYN, C. R., VAN GINNEKEN, C. J., KNAPEN, D. & VAN CRUCHTEN, S. J. 2015. Incubation at 32.5 degrees C and above causes malformations in the zebrafish embryo. *Reprod Toxicol,* 56**,** 56-63.

SAHU, A., DEVI, S., JUI, J. & GOLDMAN, D. 2021. Notch signaling via Hey1 and Id2b regulates Muller glia's regenerative response to retinal injury. *Glia,* 69**,** 2882-2898.

SONG, J., LU, Y., CHENG, X., SHI, C., LOU, Q., JIN, X., HE, J., ZHAI, G. & YIN, Z. 2021. Functions of the Thyroid-Stimulating Hormone on Key Developmental Features Revealed in a Series of Zebrafish Dyshormonogenesis Models. *Cells,* 10.

STEINER, A. B., KIM, T., CABOT, V. & HUDSPETH, A. J. 2014. Dynamic gene expression by putative hair-cell progenitors during regeneration in the zebrafish lateral line. *Proc Natl Acad Sci U S A,* 111**,** E1393-401.

VAN DINGENEN, I., ANDERSEN, E., VOLZ, S., CHRISTIANSEN, M., NOVAK, J., HAIGIS, A. C., STACY, E., BLACKWELL, B. R., VILLENEUVE, D. L., VERGAUWEN, L., HILSCHEROVA, K., HOLBECH, H. & KNAPEN, D. 2024. The thyroid hormone system disrupting potential of resorcinol in fish. *Ecotoxicol Environ Saf,* 284**,** 116995.

VANDESOMPELE, J., DE PRETER, K., PATTYN, F., POPPE, B., VAN ROY, N., DE PAEPE, A. & SPELEMAN, F. 2002. Accurate normalization of real-time quantitative RT-PCR data by geometric averaging of multiple internal control genes. *Genome Biol,* 3**,** RESEARCH0034.
